# Transcatheter vs surgical aortic valve replacement in low to intermediate surgical risk aortic stenosis patients: A systematic review and meta‐analysis of randomized controlled trials

**DOI:** 10.1002/clc.23454

**Published:** 2020-09-14

**Authors:** Dengshen Zhang, Xin Mao, Daxing Liu, Jian Zhang, Gang Luo, Liangliang Luo

**Affiliations:** ^1^ Department of Cardiovascular Surgery The Affiliated Hospital of Zunyi Medical University Zunyi Guizhou China

**Keywords:** aortic stenosis, meta‐analysis, surgical aortic valve replacement, surgical risk, transcatheter aortic valve replacement

## Abstract

**Background:**

Transcatheter aortic valve replacement (TAVR) is regarded as the most superior alternative treatment approach for patients with aortic stenosis (AS) who are associated with high surgical risk, whereas the effectiveness of TAVR vs surgical aortic valve replacement (SAVR) in low to intermediate surgical risk patients remained inconclusive. This study aimed to determine the best treatment strategies for AS with low to intermediate surgical risk based on published randomized controlled trials (RCTs).

**Hypothesis and Methods:**

RCTs that compared TAVR vs SAVR in AS patients with low to intermediate surgical risk were identified by PubMed, EmBase, and the Cochrane library from inception till April 2019. The pooled relative risks (RRs) with 95% confidence intervals (CIs) were calculated for the data collected using random‐effects models.

**Results:**

Seven RCTs with a total of 6929 AS patients were enrolled. We noted that TAVR significantly increased the risk of transient ischemic attack (TIA) (RR: 1.43; 95%CI: 1.04‐1.96; *P* = .029), and permanent pacemaker implantation (RR: 3.00; 95%CI: 1.70‐5.30; *P* < .001). However, TAVR was associated with lower risk of post‐procedural bleeding (RR: 0.57; 95%CI: 0.33‐0.98; *P* = .042), new‐onset or worsening of atrial fibrillation (RR: 0.32; 95%CI: 0.23‐0.45; *P* < .001), acute kidney injury (RR: 0.40; 95%CI: 0.25‐0.63; *P* < .001), and cardiogenic shock (RR: 0.34; 95%CI: 0.19‐0.59; *P* < .001). The risk of aortic‐valve reintervention at 1‐ (RR: 2.63; 95%CI: 1.34‐5.15; *P* = .005), and 2 years (RR: 3.19; 95%CI: 1.63‐6.24; *P* = .001) in low to intermediate surgical risk patients who received TAVR was significantly increased than those who received SAVR.

**Conclusions:**

These findings indicated that low to intermediate surgical risk patients who received TAVR had low risk of complications, whereas the risk of TIA, permanent pacemaker implantation, and aortic‐valve reintervention was increased.

## 1 INTRODUCTION

Aortic stenosis (AS) is the most frequent heart valve disease seen in elderly population, in which nearly 1/8 individuals aged 75 years or over suffer from moderate to severe AS.^1^, ^2^ The prevalence of AS in North America and Europe is 12.4%, and this implied that there are more than 291 000 candidates undergoing aortic valve replacement.^3^ The outflow of blood from the heart of AS patients is shown to be impaired, which subsequently increases cardiac workload and causes heart failure and left ventricular hypertrophy. According to a previous study, nearly 25% of mortality rates were observed annually in patients with symptomatic AS comorbidities such as angina, syncope or heart failure.^4^ So, effective treatment strategies are necessary for AS patients.

Surgical aortic valve replacement (SAVR) along with artificial prosthesis is regarded as a conventional treatment strategy due to its effective choice of intervention in operable cases with severe AS. However, very elderly patients, and patients with calcified aorta or scarring after undergoing cardiac surgery are not intolerant to SAVR. Therefore, transcatheter aortic valve replacement (TAVR) is used for inoperable or high surgical risk AS patients due to its less invasive nature.^5^, ^6^ Technological advances in valve replacement procedure produced easy repositioning and removal, and its minimally invasive nature permitted conduction of TAVR under local anesthesia, and is also associated with shorter hospital stay, low risk of bleeding and less post‐interventional complications.^7^, ^8^ Previous meta‐analyses have demonstrated that TAVR had comparable or better early and midterm outcomes in AS patients with high surgical risk.^9^, ^10^, ^11^, ^12^, ^13^ However, whether these results are suitable for low to intermediate surgical risk AS patients remains inconclusive.

Several RCTs on the research topic have been conducted, but inconsistent results were obtained from these. Clarifying optimal treatment strategy for AS patients with low to intermediate surgical risk is currently important hot spot. Therefore, a comprehensive examination of published RCTs that compared the efficacy and safety of TAVR with SAVR in low to intermediate surgical risk AS patients at various follow‐up periods was conducted.

## 2 METHODS

### 2.1 Data sources, search strategy, and selection criteria

The current study was performed according to the guidelines of preferred reporting items for systematic reviews and meta‐analysis statement.^14^ The PubMed, EmBase, and the Cochrane library were systematically searched for articles published till April 2019. The following search terms were used as medical subject headings and free‐language terms: “Transcatheter aortic valve replacement” OR “TAVR” OR “TAVI”AND “Surgical aortic valve replacement” AND “SAVR” AND “SAVI”AND “low to moderate surgical risk” AND “severe aortic stenosis”. Furthermore, trials that have been completed but not published in clinicaltrials.gov website were also searched. If essential information was unavailable from eligible publications, then corresponding authors were contacted.

The studies were searched and selected independently by two authors following a standardized flow. Disagreements between them were resolved by contacting an additional author through reviewing of the original article. The study selection process was based on PICOS criteria. The inclusion criteria were as follows: (1) patients: patients diagnosed with AS with low to intermediate surgical risk; (2) intervention: TAVR; (3) control: SAVR; (4) outcomes: the study should report at least 1 of the following outcomes: all‐cause mortality, cardiac death, stroke, transient ischemic attack (TIA), post‐procedural bleeding (PPB), permanent pacemaker implantation (PPI), new‐onset or worsening atrial fibrillation (NOWAF), acute kidney injury (AKI), major vascular complications, myocardial infarction (MI), valvular endocarditis, aortic‐valve reintervention, coronary obstruction, and cardiogenic shock; and (5) study design: studies with RCT design. Observational studies were excluded due to the possibility of confounding variables or bias in the pooled results.

### 2.2 Data collection and quality assessment

The data and quality of the included trials were collected independently by two authors following a standardized protocol, and any inconsistencies between them were settled by group discussion till a consensus was reached. The collected information was as follows: first author or study group's name, publication year, country, sample size, age, sex, society thoracic surgeons (STS) risk, logistic Euro SCORE I (LES), diabetes mellitus (DM), prior stroke, peripheral vascular disease (PVD), prior percutaneous coronary intervention (PCI), prior MI, chronic obstructive pulmonary disease (COPD), New York heart association (NYHA) III or IV, valve type, and reported outcomes. The quality of the included studies was assessed by Jadad scale based on random sequence generation, allocation concealment, blinding, intention‐to‐treat analysis, and completeness of follow‐up, and a scoring system of 0 to 5 was used for assessing the study quality.^15^

### 2.3 Statistical analysis

The investigated outcomes from each RCT were assigned as dichotomous data, and the relative risks (RRs) and 95% confidence intervals (CIs) were calculated by using the event number extracted from each trial before data pooling. After this, the summary RRs and 95%CIs for investigated outcomes were calculated using random‐effects model as the true effect that underlies varies among the included trials.^16^, ^17^ Heterogeneity was assessed by using I‐square and Q statistics across the included trials, and *P* < .10 was considered as statistically significant heterogeneity.^18^, ^19^ Sensitivity analyses were conducted for studies that reported outcomes ≥5 to assess the impact of single study from overall analyses.^20^ Moreover, subgroup analyses were conducted for studies that reported outcomes ≥5 based on sample size, mean age, STS score, percentage of DM, prior stroke, prior PVD, prior MI, prior COPD, percentage of NYHA III‐IV, valve type, follow‐up duration, and study quality. After this, *P* values between subgroups were calculated using interaction tests, which were based on Student's *t* distribution.^21^ Publication biases for studies that reported outcomes ≥5 were assessed by funnel plots, Egger,^22^ and Begg tests.^23^ The *P* values for pooled results are two‐sided, and *P* < .05 was regarded as statistically significant. All statistical analyses in this study were conducted using STATA software version 10.0 (Stata Corp., Texas).

## 3 RESULTS

### 3.1 Literature search

**FIGURE 1:**
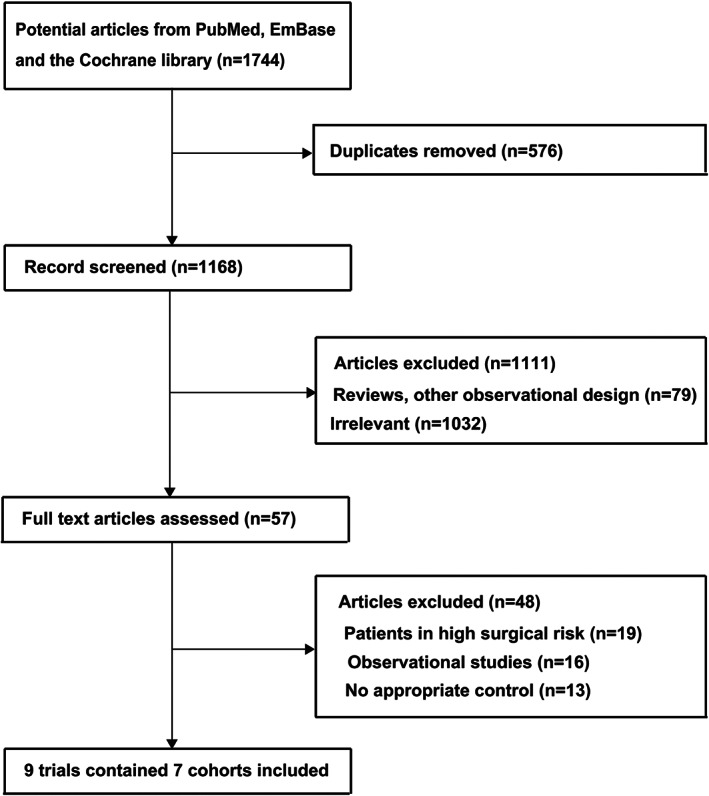
Flow diagram of literature search and trials selection process

In total, 1744 articles were retrieved from the initial search. Of these, 1687 were excluded after reviewing the titles and abstracts due to duplications or irrelevant topics. Further detailed evaluation was performed for the remaining 57 studies, and finally nine studies that reported seven cohorts were selected for this meta‐analysis.^24^, ^25^, ^26^, ^27^, ^28^, ^29^, ^30^ No additional eligible study was retrieved by manual searching of the references of relevant studies. The details of study selection process was shown as flowchart and listed in Figure 1.

### 3.2 Study characteristics

The general characteristics of included studies and patients were summarized in Table 1, and these studies were published from 2012 to 2019. A total of 6929 AS patients with low to intermediate surgical risk from seven cohorts were included, and patients ranged from 70 to 2032 in each individual study. The mean age of these patients ranged from 73.4 to 81.6 years, and the percentage of males ranged from 30.0% to 69.3%. Moreover, the STS score of enrolled patients ranged from 1.9 to 5.8. Three cohorts reported patients who received balloon‐expanding TAVR, and the remaining four cohorts included patients who received self‐expanding TAVR. Study quality was assessed by JADAD scale, in which four cohorts have scored four, and the remaining three cohorts have scored three.

**TABLE 1:** Baseline characteristics of studies included in the systematic review and meta‐analysis

Study	Country	Sample size	Age (years)	Male	STS	LES	DM	Prior stroke	PVD	Prior PCI	Prior MI	COPD	NYHA III‐IV	Valve type	JADAD score
Nielsen (STACCATO 2012)^25^	Nordic region	70	81.0	21 (30.0%)	3.3	9.9	4 (5.7%)	2 (2.9%)	5 (7.1%)	NA	NA	2 (2.9%)	NA	Balloon‐expanding	3
Thyregod (NOTION 2015)^26^, ^27^	Denmark and Sweden	280	79.1	149 (53.2%)	3.0	8.6	54 (19.3%)	46 (16.4%)	15 (5.4%)	23 (8.2%)	14 (5.0%)	33 (11.8%)	47.1%	Self‐expanding	3
Reardon (CoreValve US 2016)^28^	United States	383	81.4	218 (56.9%)	5.3	NA	NA	NA	NA	NA	NA	NA	NA	Self‐expanding	3
Leon (PARTNER 22016)^29^	United States and Canada	2032	81.6	1108 (54.5%)	5.8	NA	730 (35.9%)	642 (31.6%)	618 (30.4%)	556 (27.4%)	364 (15.6%)	627 (30.9%)	76.7%	Balloon‐expanding	4
Reardon (SURTAVI 2017)^30^, ^31^	United States, The Netherlands, Germany, UK, Spain, Switzerland, Sweden, Canada, Denmark	1746	79.9	992 (56.8%)	4.4	11.8	592 (33.9%)	124 (7.1%)	533 (30.5%)	369 (21.1%)	241 (13.8%)	NA	58.9%	Self‐expanding	4
Popma (Evolut Low Risk Trial 2019)^32^	Australia, Canada, France, Japan, the Netherlands, New Zealand, and the United States	1468	73.9	956 (65.1%)	1.9	NA	452 (30.8%)	158 (10.8%)	117 (8.0%)	195 (13.3%)	88 (6.0%)	227 (15.5%)	26.3%	Self‐expanding	4
Mack (PARTNER 32019)^33^	United States, Germany, Canada, and UK	950	73.4	658 (69.3%)	1.9	NA	292 (30.7%)	40 (4.2%)	67 (7.1%)	NA	54 (5.7%)	53 (5.6%)	27.7%	Balloon‐expanding	4

Abbreviations: DM, diabetes mellitus; LES, logistic EuroSCORE I; MI, myocardial infarction; NYHA, New York heart association; PCI, percutaneous coronary intervention; PVD, peripheral vascular disease; STS, society thoracic surgeons risk.

### 3.3 All‐cause mortality and cardiac death

**FIGURE 2:**
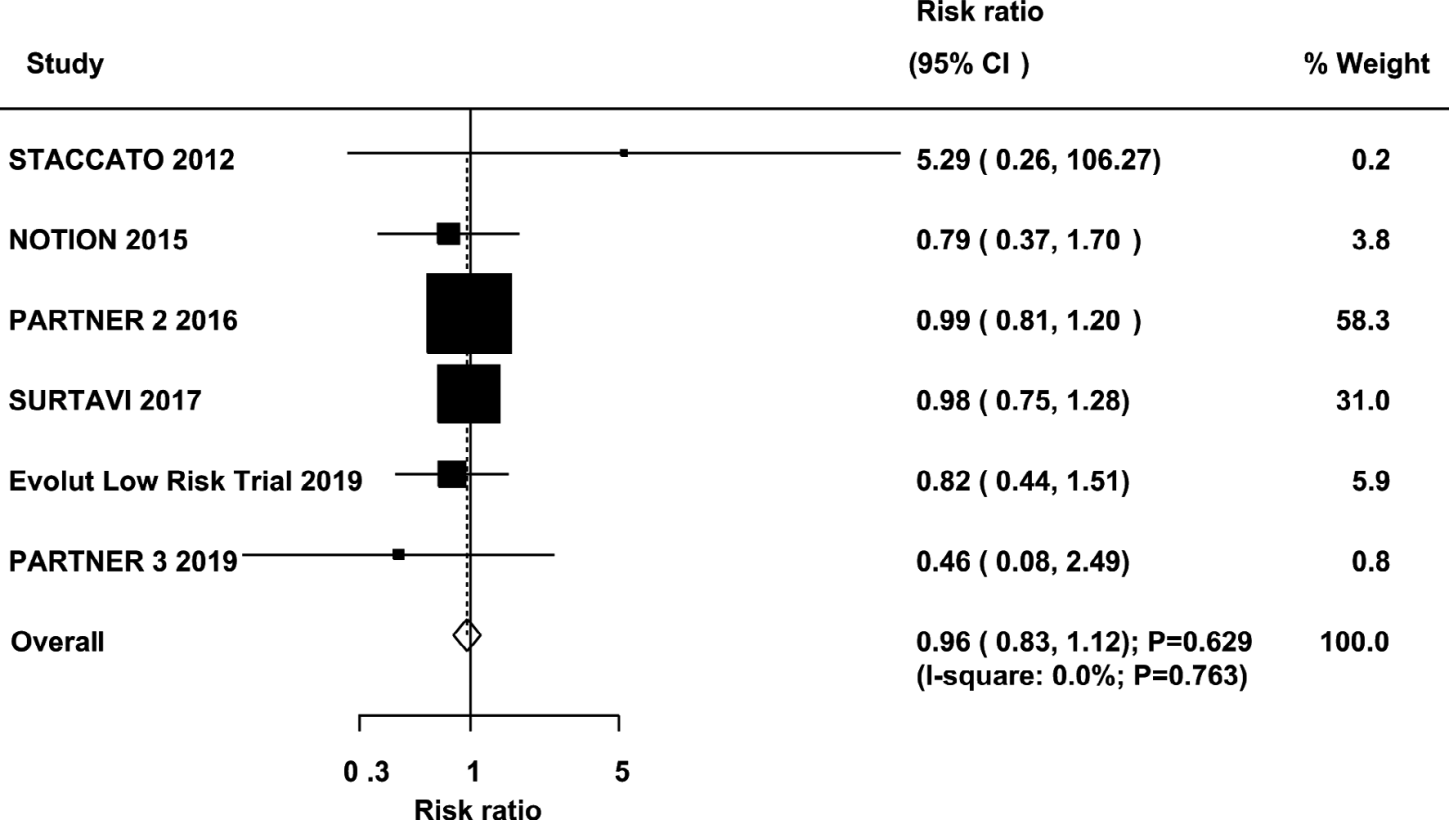
TAVR versus SAVR on the risk of all‐cause mortality. SAVR, surgical aortic valve replacement; TAVR, transcatheter aortic valve replacement

**FIGURE 3:**
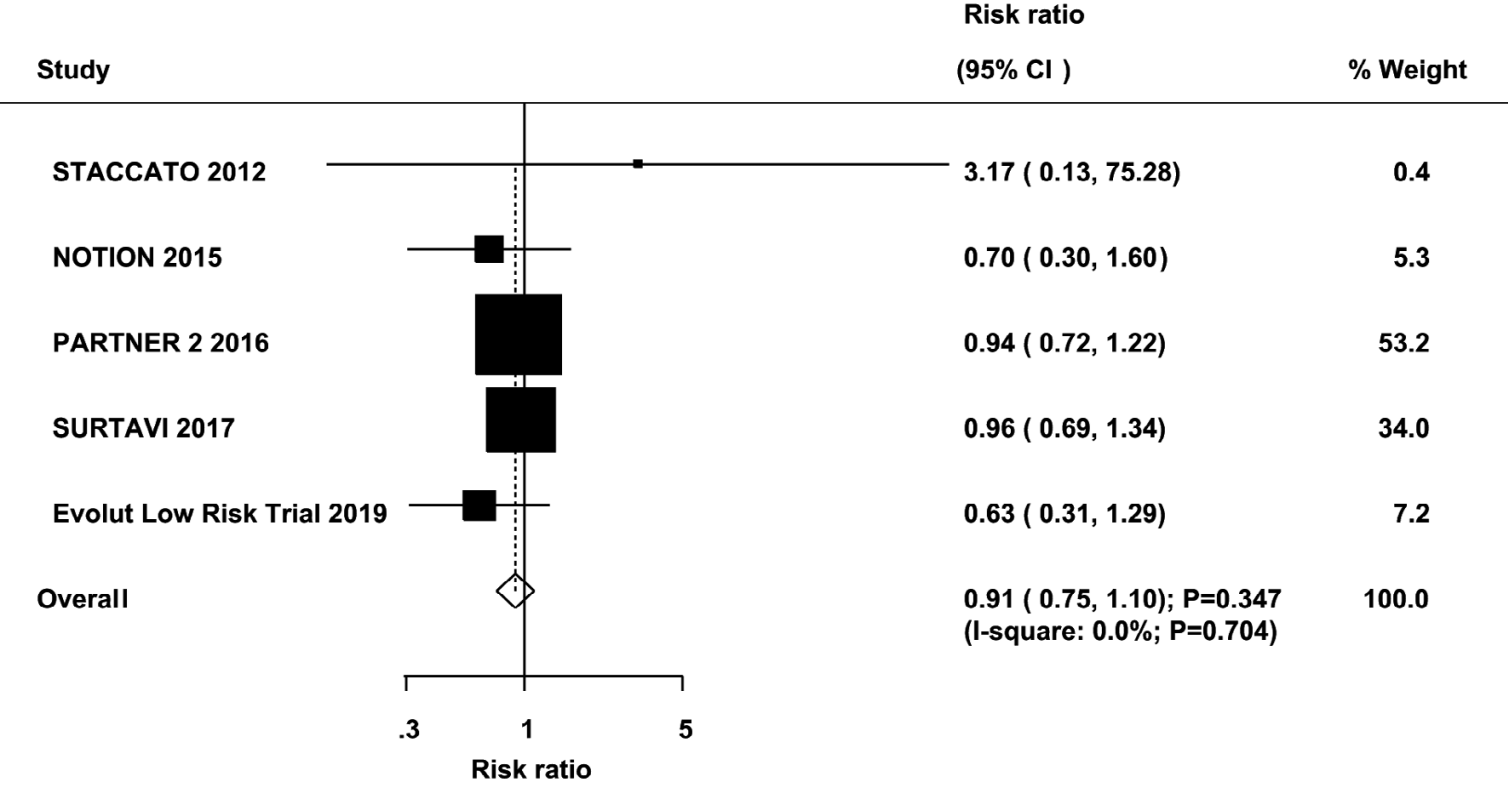
TAVR versus SAVR on the risk of cardiac death. SAVR, surgical aortic valve replacement; TAVR, transcatheter aortic valve replacement

Data regarding the effect of TAVR vs SAVR on the risk of all‐cause mortality and cardiac death were available in six and five cohorts, respectively. There were no significant differences between TAVR and SAVR on the risk of all‐cause mortality (RR: 0.96; 95%CI: 0.83‐1.12; *P* = .629; with no evidence of heterogeneity; Figure 2) and cardiac death (RR: 0.91; 95%CI: 0.75‐1.10; *P* = .347; with unimportant heterogeneity; Figure 3). Sensitivity analyses indicated that the risk of all‐cause mortality and cardiac death between TAVR and SAVR remained stable (Supplemental Figures 13 and 15). No significant differences for all‐cause mortality and cardiac death were detected by subgroup analyses (Supplemental Tables 1 and 2). No significant publication bias for all‐cause mortality and cardiac death was detected (Supplemental Figures 14 and 16).

### 3.4 Stroke and TIA

Data regarding the effect of TAVR vs SAVR on the risk of stroke and TIA were available in seven and five cohorts, respectively. The results showed that TAVR was not associated with the risk of stroke (RR: 0.85; 95%CI: 0.67‐1.09; unimportant heterogeneity; Supplemental Figure 1), whereas the risk of TIA was significantly increased in patients who received TAVR (RR: 1.43; 95%CI: 1.04‐1.96; *P* = .029; with no evidence of heterogeneity; Supplemental Figure 2). Sensitivity analysis indicated that TAVR might protect against the risk of stroke if PARTNER 2 cohort was excluded,^26^ as it included high prevalence of stroke history (31.6%) and caused high risk of recurrent stroke (Supplemental Figure 17). The risk of TIA was unstable due to marginal 95%CI when sensitivity analysis was conducted (Supplemental Figure 19). Subgroup analysis indicated no significant risk on stroke in all the subsets, whereas increased risk of TIA was mainly observed in mean age of patients ≥80.0 years, STS score ≥ 3.5, prior PVD ≥10.0%, prior MI ≥10.0%, percentage of NYHA III‐IV ≥50.0%, patients who received balloon‐expanding TAVR, and followed up for 2 years (Supplemental Tables 3 and 4). No evidence of publication bias for stroke and TIA was detected (Supplemental Figures 18 and 20).

### 3.5 PPB and PPI

Data regarding the effect of TAVR vs SAVR on the risk of PPB and PPI were available in six and six cohorts, respectively. The results of TAVR showed association with reduced risk of PPB (RR: 0.57; 95%CI: 0.33‐0.98; *P* = .042; Supplemental Figure 3), while the incidence of PPI showed significant increase (RR: 3.00; 95%CI: 1.70‐5.30; *P* < .001; Supplemental Figure 4). Sensitivity analyses indicated that the risk of PPB between TAVR and SAVR varied, while for the risk of PPI was stable (Supplemental Figures 21 and 23). Subgroup analyses indicated that the reduced risk of PPB was mainly detected when pooling the studies with sample size <1000, mean age of patients ≥80.0 years, STS score < 3.5, percentage of DM < 30.0% or not reported, prior stroke ≥10.0% or not reported, prior PVD <10.0% or not reported, prior MI < 10.0% or not reported, prior COPD ≥10.0%, percentage of NYHA III‐IV < 50.0% or not reported, patients who received balloon‐expanding TAVR, followed up for 1 and 2 years, and studies with low quality (Supplemental Table 5). Moreover, no significant effect on PPI was observed if the mean age of patients ≥80.0 years, prior PVD ≥10.0%, prior MI ≥10.0%, prior COPD <10.0%, percentage of NYHA III‐IV ≥50.0%, and patients who received balloon‐expanding TAVR (Supplemental Table 6). No significant publication bias for PPB and PPI was observed (Supplemental Figures 22 and 24).

### 3.6 NOWAF and AKI

Data regarding the effect of TAVR vs SAVR on the risk of NOWAF and AKI were available in 6 and 6 cohorts, respectively. The results revealed that TAVR significantly reduced the risk of NOWAF (RR: 0.32; 95%CI: 0.23‐0.45; *P* < .001; significant heterogeneity; Supplemental Figure 5) and AKI (RR: 0.40; 95%CI: 0.25‐0.63; *P* < .001; moderate heterogeneity; Supplemental Figure 6). Sensitivity analyses indicated pooled conclusion of NOWAF and AKI was robust and unaltered by excluding any particular study (Supplemental Figures 25 and 27). The risk of NOWAF was significantly reduced in all subsets, whereas no significant difference on the risk of AKI when the sample size <1000, mean age of patients ≥80.0 years, STS score < 3.5, percentage of DM < 30.0%, prior stroke <10.0%, prior PVD < 10.0%, studies that did not report prior MI, prior COPD <10.0%, studies that did not report percentage of NYHA III‐IV, followed up till 1 year, and studies with low quality (Supplemental Tables 7 and 8). Finally, no evidence of publication bias for NOWAF and AKI was observed (Supplemental Figures 26 and 28).

### 3.7 Major vascular complications

Data regarding the effect of TAVR vs SAVR on the risk of major vascular complications were available in five cohorts. Overall, there was no significant difference between TAVR and SAVR on the risk of major vascular complications (RR: 1.41; 95%CI: 0.96‐2.06; *P* = .080; Supplemental Figure 7), and observed significant heterogeneity across the included studies. Sensitivity analysis indicated that TAVR might increase the risk of major vascular complications after excluding SURTAVI study (Supplemental Figure 29).^30^, ^31^ Subgroup analyses indicated that TAVR was associated with greater risk of major vascular complications if sample size <1000, studies with no percentage of DM, studies that reported no prior stroke, studies that did not report prior PVD, studies that did not report prior MI, studies that did not report the percentage of NYHA III‐IV, followed up for 30 days, and studies with low quality (Supplemental Table 9). Significant publication bias was inevident (*P* value for Egger: 0.118; *P* value for Begg: 0.462; Supplemental Figure 30).

### 3.8 Myocardial infarction

The number of cohorts available for MI follow‐up at 30 days, 1 year, and 2 years were four, four, and three cohorts, respectively. The results revealed that TAVR showed no association with the risk of MI when compared to SAVR follow‐up at 30 days (RR: 0.67; 95%CI: 0.43‐1.06; *P* = .089; without evidence of heterogeneity), 1 year (RR: 0.91; 95%CI: 0.64‐1.30; *P* = .616; without evidence of heterogeneity), and 2 years (RR: 0.98; 95%CI: 0.70‐1.39; *P* = .926; without evidence of heterogeneity) (Supplemental Figure 8).

### 3.9 Valvular endocarditis

The breakdown regarding the number of cohorts available for valvular endocarditis follow‐up at 30 days, 1 year, and 2 years were two, three, and one cohorts, respectively. No significant differences between TAVR and SAVR with regard to the risk of valvular endocarditis follow‐up were observed at 30 days (RR: 1.55; 95%CI: 0.19‐12.60; *P* = .679; without evidence of heterogeneity), 1 year (RR: 1.11; 95%CI: 0.48‐2.58; *P* = .811; without evidence of heterogeneity), and 2 years (RR: 1.85; 95%CI: 0.69‐4.99; *P* = .223) (Supplemental Figure 9).

### 3.10 Aortic‐valve reintervention

The breakdown regarding the number of cohorts available for aortic‐valve reintervention follow‐up at 30 days, 1 year, and 2 years were three, three, and two cohorts, respectively. The results revealed that TAVR showed no association with the risk of aortic‐valve reintervention follow‐up for 30 days (RR: 2.43; 95%CI: 0.78‐7.60; *P* = .126; with unimportant heterogeneity), whereas TAVR significantly increased the risk of aortic‐valve reintervention follow‐up at 1 year (RR: 2.63; 95%CI: 1.34‐5.15; *P* = .005; without evidence of heterogeneity), and 2 years (RR: 3.19; 95%CI: 1.63‐6.24; *P* = .001; without evidence of heterogeneity) (Supplemental Figure 10).

### 3.11 Coronary obstruction

The number of cohorts available for coronary obstruction follow‐up at 30 days, 1 year, and 2 years were three, two, and one cohorts, respectively. TAVR and SAVR on the risk of coronary obstruction follow‐up at 30 days (RR: 1.40; 95%CI: 0.51‐3.85; *P* = .517; with unimportant heterogeneity), 1 year (RR: 1.22; 95%CI: 0.36‐4.14; *P* = .745; with moderate heterogeneity), and 2 years (RR: 0.67; 95%CI: 0.19‐2.38; *P* = .539) showed no significant differences (Supplemental Figure 11).

### 3.12 Cardiogenic shock

Data on the effect of TAVR on the risk of cardiogenic shock at 30 days follow‐up were available in two studies. The results showed that patients who received TAVR had a reduced risk of cardiogenic shock than those who received SAVR (RR: 0.34; 95%CI: 0.19‐0.59; *P* < .001; without evidence of heterogeneity; Supplemental Figure 12).

## 4 DISCUSSION

The current study included seven RCTs and used meta‐analysis to provide solid supporting evidence. The summary results of this study indicated that TAVR demonstrated beneficial effects of PPB, NOWAF, AKI, and cardiogenic shock, whereas TAVR produced excess risk of TIA, PPI, and aortic‐valve reintervention when compared with SAVR. Finally, TAVR and SAVR showed no significant differences on the risk of all‐cause mortality, cardiac death, stroke, major vascular complications, MI, valvular endocarditis, and coronary obstruction.

Numerous systematic reviews and meta‐analyses were conducted on this topic; however, there are several inherent limitations in these studies^31^, ^32^, ^33^, ^34^, ^35^, ^36^, ^37^, ^38^(Supplemental Table 10). Two studies showed association of TAVR with reduced risk of mortality,^31^, ^32^ while the remaining six studies showed no significant difference between TAVR and SAVR on the risk of mortality.^33^, ^34^, ^35^, ^36^, ^37^, ^38^ The risk of cardiac death between TAVR and SAVR showed no significant association.^32^, ^38^ Three studies found association of TAVR with reduced risk of stroke,^31^, ^35^, ^38^ while one study showed increased risk of stroke in patients with TAVR.^33^ Similarly, this study reported inconsistent results on the risk of PPB. Furthermore, the risk of PPI, AKI, and major vascular complications between TAVR and SAVR across prior meta‐analyses was consistent. However, the results of previous studies contained both RCTs as well as observational studies, inducing uncontrolled confounders and causing over estimation of pooled results. Moreover, the results of these studies were stratified based on study design and follow‐up duration, and whether the effects of treatment differed according to the characteristics of patients were not illustrated. Moreover, a meta‐analysis conducted by Ando et al reported the association of TAVR with reduced risk of all‐cause mortality or disabling/major stroke at 1 year as compared with SAVR in patients with low to intermediate surgical risk.^39^ However, the study did not report other endpoints, which requires evaluation through pooling of all published RCTs. Therefore, the current study was conducted based on published RCTs to determine the effect of TAVR vs SAVR in AS patients with low to intermediate surgical risk.

The summary results of this study reported that TAVR vs SAVR revealed no association with the risk of all‐cause mortality and cardiac death. These results were stable and showed no significant differences through sensitivity and subgroup analyses. This suggested that an additional TAVR specific risk model should be constructed for better stratification.^40^ Furthermore, the summary results indicated that the risk of stroke, major vascular complications, and MI between TAVR and SAVR showed no significant association, while the risk of TIA in TAVR group was significantly increased. However, the risk of stroke might be biased by the PARTNER 2 cohort,^26^ as this study specifically included patients with high STS score, and high prevalence of prior stroke. Moreover, the risk of TIA was consistent with PARTNER two cohort,^26^ which was 3.7% and 2.3% in TAVR and SAVR groups. This significantly increased the risk mainly in elderly patients, with high STS score, high prior PVD, MI, or NYHA III‐IV, who received balloon‐expanding TAVR, and had longer follow‐up duration. These results suggested that high risk score patients with low to intermediate surgical risk should avoid the use of TAVR. However, the incidence of TIA was lower and the results should be further verified in RCTs with longer follow‐up duration.

TAVR showed association with less complications post‐procedurally, which included bleeding, NOWAF, AKI, and cardiogenic shock, and was consistent with previous meta‐analyses findings.^34^, ^35^, ^37^ Moreover, the risk of PPI and aortic‐valve reintervention was shown to be significantly higher in TAVR group. Patients who received TAVR with high risk of conduction disturbances could explain these increased risk factors.^41^ These reduced the risk that could be explained by minimally invasive approach when compared with traditional SAVR. Furthermore, the groups with the risk of valvular endocarditis and coronary obstruction showed no significant differences. However, these results were unstable as these outcomes were reported by smaller number of cohorts, which in turn produce broad confidence intervals, with no statistically significant differences.

However, there are several limitations in this study that should be acknowledged: (1) Some studies might have been missed as they were not included in the searched databases, and this might in turn produce inevitable publication bias; (2) a smaller number of cohorts were included in some subgroups, inducing variable results; (3) there might be bias due to TAVR, which induced substantial heterogeneity and affect treatment effectiveness of TAVR; (4) the causes of aortic valve re‐intervention were not available from the included trials; (5) the treatment strategies after TAVR or SAVR were not available across the included studies, which could affect the prognosis of low to intermediate surgical risk AS patients; and (6) this study analysis was based on pooled data, restricting us from conducting a more detailed analysis.

## 5 CONCLUSION

In conclusion, this study demonstrated similar prevalence of all‐cause mortality, cardiac death, stroke, major vascular complications, MI, valvular endocarditis, and coronary obstruction between both approaches of TAVR and SAVR in low to intermediate surgical risk AS patients. However, TAVR induced a greater risk of TIA, PPI, and aortic‐valve reintervention, and protected against the risk of PPB, NOWAF, AKI, and cardiogenic shock. Further large‐scale RCTs should be conducted to verify the results of subgroup analyses.

## CONFLICT OF INTERESTS

All authors declare that they have no any conflict of interests.

## Supporting information


**Figure S1** TAVR vs SAVR on the risk of stroke
**Figure S2**. TAVR vs SAVR on the risk of transient ischemic attack
**Figure S3**. TAVR vs SAVR on the risk of post‐procedural bleeding
**Figure S4**. TAVR vs SAVR on the risk of permanent pacemarker implantation
**Figure S5**. TAVR vs SAVR on the risk of new‐onset or worsening atrial fibrillation
**Figure S6**. TAVR vs SAVR on the risk of acute kidney injury
**Figure S7**. TAVR vs SAVR on the risk of major vascular complications
**Figure S8**. TAVR vs SAVR on the risk of myocardial infarction
**Figure S9**. TAVR vs SAVR on the risk of valvular endocarditis
**Figure S10** TAVR vs SAVR on the risk of aortic‐valve reintervention
**Figure S11** TAVR vs SAVR on the risk of coronary obstruction
**Figure S12** TAVR vs SAVR on the risk of cardiogenic shock.
**Figure S13** Sensitivity analysis for all‐cause mortality
**Figure S14** Funnel plot for all‐cause mortality
**Figure S15** Sensitivity analysis for cardiac death
**Figure S16** Funnel plot for cardiac death
**Figure S17** Sensitivity analysis for stroke
**Figure S18** Funnel plot for stroke
**Figure S19** Sensitivity analysis for TIA
**Figure S20** Funnel plot for TIA
**Figure S21** Sensitivity analysis for post‐procedural bleeding
**Figure S22** Funnel plot for post‐procedural bleeding
**Figure S23**. Sensitivity analysis for permanent pacemarker implatation
**Figure S24** Funnel plot for permanent pacemarker implatation
**Figure S25** Sensitivity analysis for new‐onset or worsening atrial fibrillation
**Figure S26** Funnel plot for new‐onset or worsening atrial fibrillation
**Figure S27** Sensitivity analysis for AKI
**Figure S28** Funnel plot for AKI
**Figure S29** Sensitivity analysis for major vascular complications
**Figure S30** Funnel plot for major vascular complications

**Table S1** Subgroup analysis for all‐cause mortality
**Table S2**. Subgroup analysis for cardiac death
**Table S3**. Subgroup analysis for stroke
**Table S4**. Subgroup analysis for TIA
**Table S5**. Subgroup analysis for post‐procedural bleeding
**Table S6**. Subgroup analysis for permanent pacemarker implatation
**Table S7**. Subgroup analysis for new‐onset or worsening atrial fibrillation
**Table S8**. Subgroup analysis for acute kidney injury
**Table S9**. Subgroup analysis for major vascular complications
**Table S10**. The results of previous meta‐analyses

## References

[1] Nkomo VT , Gardin JM , Skelton TN , Gottdiener JS , Scott CG , Enriquez‐Sarano M . Burden of valvular heart diseases: a population‐based study. Lancet. 2006;368:1005‐1011. 16980116 10.1016/S0140-6736(06)69208-8

[2] Lindroos M , Kupari M , Heikkila J , et al. Prevalence of aortic valve abnormalities in the elderly: an echocardiographic study of a random population sample. J Am Coll Cardiol. 1993;21:1220‐1225. 8459080 10.1016/0735-1097(93)90249-z

[3] Osnabrugge RL , Mylotte D , Head SJ , et al. Aortic stenosis in the elderly: disease prevalence and number of candidates for transcatheter aortic valve replacement: a meta‐analysis and modeling study. J Am Coll Cardiol. 2013;62:1002‐1012. 23727214 10.1016/j.jacc.2013.05.015

[4] Carabello BA , Paulus WJ . Aortic stenosis. Lancet. 2009;373:956‐966. 19232707 10.1016/S0140-6736(09)60211-7

[5] Nishimura RA , Otto CM , Bonow RO , et al. AHA/ACC focused update of the 2014 AHA/ACC guideline for the management of patients with valvular heart disease: a report of the American college of cardiology/American heart association task force on clinical practice guidelines. Circulation. 2017;135:e1159‐e1195. 28298458 10.1161/CIR.0000000000000503

[6] Vahanian A , Alfieri O , Andreotti F , et al. Guidelines on the management of valvular heart disease (version 2012). Eur Heart J. 2012;33:2451‐2496. 22922415 10.1093/eurheartj/ehs109

[7] Oguri A , Yamamoto M , Mouillet G , et al. Clinical outcomes and safety of transfemoral aortic valve implantation under general versus local anesthesia: subanalysis of the French aortic national CoreValve and Edwards 2 registry. Circ Cardiovasc Interv. 2014;7:602‐610. 25006175 10.1161/CIRCINTERVENTIONS.113.000403

[8] Rees CM , Eric HO . Should patients with low‐moderate surgical risk be offered TAVI instead of conventional aortic valve replacement in the management of symptomatic aortic stenosis? Res Medica. 2015;23:15‐21.

[9] Liu Z , Kidney E , Bem D , et al. Transcatheter aortic valve implantation for aortic stenosis in high surgical risk patients: a systematic review and meta‐analysis. PLoS One. 2018;13:e0196877. 29746546 10.1371/journal.pone.0196877 PMC5944928

[10] Villablanca PA , Mathew V , Thourani VH , et al. A meta‐analysis and meta‐regression of long‐term outcomes of transcatheter versus surgical aortic valve replacement for severe aortic stenosis. Int J Cardiol. 2016;225:234‐243. 27732927 10.1016/j.ijcard.2016.10.003

[11] Siontis GC , Praz F , Pilgrim T , et al. Transcatheter aortic valve implantation vs. surgical aortic valve replacement for treatment of severe aortic stenosis: a meta‐analysis of randomized trials. Eur Heart J. 2016;37:3503‐3512. 27389906 10.1093/eurheartj/ehw225

[12] Gargiulo G , Sannino A , Capodanno D , et al. Transcatheter aortic valve implantation versus surgical aortic valve replacement: a systematic review and meta‐analysis. Ann Intern Med. 2016;165:334‐344. 27272666 10.7326/M16-0060

[13] Takagi H , Niwa M , Mizuno Y , Goto SN , Umemoto T , All‐Literature Investigation of Cardiovascular Evidence (ALICE) Group . A meta‐analysis of transcatheter aortic valve implantation versus surgical aortic valve replacement. Ann Thorac Surg. 2013;96:513‐519. 23816417 10.1016/j.athoracsur.2013.04.049

[14] Moher D , Liberati A , Tetzlaff J , Altman DG , The PRISMA Group . Preferred reporting items for systematic reviews and meta‐analyses: the PRISMA statement. PLoS Med. 2009;6:e1000097. 19621072 10.1371/journal.pmed.1000097 PMC2707599

[15] Jadad AR , Moore RA , Carroll D , et al. Assessing the quality of reports of randomized clinical trials: is blinding necessary? Control Clin Trials. 1996;17:1‐12. 8721797 10.1016/0197-2456(95)00134-4

[16] DerSimonian R , Laird N . Meta‐analysis in clinical trials. Control Clin Trials. 1986;7:177‐188. 3802833 10.1016/0197-2456(86)90046-2

[17] Ades AE , Lu G , Higgins JP . The interpretation of random‐effects meta‐analysis in decision models. Med Decis Making. 2005;25:646‐654. 16282215 10.1177/0272989X05282643

[18] Deeks JJ , Higgins JPT , Altman DG . Analyzing data and undertaking meta‐analyses. Oxford, UK: The Cochrane Collaboration; 2008.

[19] Higgins JP , Thompson SG , Deeks JJ , Altman DG . Measuring inconsistency in meta‐analyses. BMJ. 2003;327:557‐560. 12958120 10.1136/bmj.327.7414.557 PMC192859

[20] Tobias A . Assessing the influence of a single study in meta‐analysis. Stata Tech Bull. 1999;47:15‐17.

[21] Altman DG , Bland JM . Interaction revisited: the difference between two estimates. BMJ. 2003;326:219. 12543843 10.1136/bmj.326.7382.219 PMC1125071

[22] Egger M , Davey Smith G , Schneider M , et al. Bias in meta‐analysis detected by a simple, graphical test. BMJ. 1997;315:629‐634. 9310563 10.1136/bmj.315.7109.629 PMC2127453

[23] Begg CB , Mazumdar M . Operating characteristics of a rank correlation test for publication bias. Biometrics. 1994;50:1088‐1101. 7786990

[24] Sondergaard L , Steinbruchel DA , Ihlemann N , et al. Two‐year outcomes in patients with severe aortic valve stenosis randomized to Transcatheter versus surgical aortic valve replacement: the all‐comers Nordic aortic valve intervention randomized clinical trial. Circ Cardiovasc Interv. 2016;9(6):e003665. 27296202 10.1161/CIRCINTERVENTIONS.115.003665

[25] Reardon MJ , Kleiman NS , Adams DH , et al. Outcomes in the randomized CoreValve US pivotal high risk trial in patients with a Society of Thoracic Surgeons risk score of 7% or less. JAMA Cardiol. 2016;1:945‐949. 27541162 10.1001/jamacardio.2016.2257

[26] Leon MB , Smith CR , Mack MJ , et al. Transcatheter or surgical aortic‐valve replacement in intermediate‐risk patients. N Engl J Med. 2016;374:1609‐1620. 27040324 10.1056/NEJMoa1514616

[27] Reardon MJ , Van Mieghem NM , Popma JJ , et al. Surgical or Transcatheter aortic‐valve replacement in intermediate‐risk patients. N Engl J Med. 2017;376:1321‐1331. 28304219 10.1056/NEJMoa1700456

[28] Serruys PW , Modolo R , Reardon M , et al. One‐year outcomes of patients with severe aortic stenosis and an STS PROM of less than three percent in the SURTAVI trial. EuroIntervention. 2018;14:877‐883. 29992904 10.4244/EIJ-D-18-00460

[29] Popma JJ , Deeb GM , Yakubov SJ , et al. Transcatheter aortic‐valve replacement with a self‐expanding valve in low‐risk patients. N Engl J Med. 2019;380:1706‐1715. 30883053 10.1056/NEJMoa1816885

[30] Mack MJ , Leon MB , Thourani VH , et al. Transcatheter aortic‐valve replacement with a balloon‐expandable valve in low‐risk patients. N Engl J Med. 2019;380:1695‐1705. 30883058 10.1056/NEJMoa1814052

[31] Arora S , Strassle PD , Ramm CJ , et al. Transcatheter versus surgical aortic valve replacement in patients with lower surgical risk scores: a systematic review and meta‐analysis of early outcomes. Heart Lung Circ. 2017;26:840‐845. 28169084 10.1016/j.hlc.2016.12.003

[32] Singh K , Carson K , Rashid MK , et al. Transcatheter aortic valve implantation in intermediate surgical risk patients with severe aortic stenosis: a systematic review and meta‐analysis. Heart Lung Circ. 2018;27:227‐234. 28473216 10.1016/j.hlc.2017.02.032

[33] Khan AR , Khan S , Riaz H , et al. Efficacy and safety of transcatheter aortic valve replacement in intermediate surgical risk patients: a systematic review and meta‐analysis. Catheter Cardiovasc Interv. 2016;88:934‐944. 26946091 10.1002/ccd.26465

[34] Khan SU , Lone AN , Saleem MA , Kaluski E . Transcatheter vs surgical aortic‐valve replacement in low‐ to intermediate‐surgical‐risk candidates: a meta‐analysis and systematic review. Clin Cardiol. 2017;40:974‐981. 29168984 10.1002/clc.22807 PMC6490337

[35] Tam DY , Vo TX , Wijeysundera HC , et al. Transcatheter vs surgical aortic valve replacement for aortic stenosis in low‐intermediate risk patients: a meta‐analysis. Can J Cardiol. 2017;33:1171‐1179. 28843328 10.1016/j.cjca.2017.06.005

[36] Elmaraezy A , Ismail A , Abushouk AI , et al. Efficacy and safety of transcatheter aortic valve replacement in aortic stenosis patients at low to moderate surgical risk: a comprehensive meta‐analysis. BMC Cardiovasc Disord. 2017;17:234. 28836953 10.1186/s12872-017-0668-1 PMC5571502

[37] Garg A , Rao SV , Visveswaran G , et al. Transcatheter aortic valve replacement versus surgical valve replacement in low‐intermediate surgical risk patients: a systematic review and meta‐analysis. J Invasive Cardiol. 2017;29:209‐216. 28570236

[38] Zhou Y , Wang Y , Wu Y , Zhu J . Transcatheter versus surgical aortic valve replacement in low to intermediate risk patients: a meta‐analysis of randomized and observational studies. Int J Cardiol. 2017;228:723‐728. 27886617 10.1016/j.ijcard.2016.11.262

[39] Ando T , Ashraf S , Villablanca P , et al. Meta‐analysis of effectiveness and safety of Transcatheter aortic valve implantation versus surgical aortic valve replacement in low‐to‐intermediate surgical risk cohort. Am J Cardiol. 2019;124:580‐585. 31200922 10.1016/j.amjcard.2019.05.017

[40] Wang TKM , Wang MTM , Gamble GD , Webster M , Ruygrok PN . Performance of contemporary surgical risk scores for transcatheter aortic valve implantation: a meta‐analysis. Int J Cardiol. 2017;236:350‐355. 28111053 10.1016/j.ijcard.2016.12.188

[41] Siontis GC , Juni P , Pilgrim T , et al. Predictors of permanent pacemaker implantation in patients with severe aortic stenosis undergoing TAVR: a meta‐analysis. J Am Coll Cardiol. 2014;64:129‐140. 25011716 10.1016/j.jacc.2014.04.033

