# Supplementary material for: Transcatheter vs surgical aortic valve replacement in low to intermediate surgical risk aortic stenosis patients: A systematic review and meta‐analysis of randomized controlled trials
Source: Clin Cardiol. 2020 Sep 14;43(12):1414–22. doi: 10.1002/clc.23454 (PMC7724228; doi:10.1002/clc.23454)
Supplement: Supplementary file 1 — Figure S1 TAVR vs SAVR on the risk of stroke Figure S2. TAVR vs SAVR on the risk of transient ischemic attack Figure S3. TAVR vs SAVR on the risk of post‐procedural bleeding Figure S4. TAVR vs SAVR on the risk of permanent pacemarker implantation Figure S5. TAVR vs SAVR on the risk of new‐onset or worsening atrial fibrillation Figure S6. TAVR vs SAVR on the risk of acute kidney injury Figure S7. TAVR vs SAVR on the risk of major vascular complications Figure S8. TAVR vs SAVR on the risk of myocardial infarction Figure S9. TAVR vs SAVR on the risk of valvular endocarditis Figure S10 TAVR vs SAVR on the risk of aortic‐valve reintervention Figure S11 TAVR vs SAVR on the risk of coronary obstruction Figure S12 TAVR vs SAVR on the risk of cardiogenic shock. Figure S13 Sensitivity analysis for all‐cause mortality Figure S14 Funnel plot for all‐cause mortality Figure S15 Sensitivity analysis for cardiac death Figure S16 Funnel plot for cardiac death Figure S17 Sensitivity analysis for stroke Figure S18 Funnel plot for stroke Figure S19 Sensitivity analysis for TIA Figure S20 Funnel plot for TIA Figure S21 Sensitivity analysis for post‐procedural bleeding Figure S22 Funnel plot for post‐procedural bleeding Figure S23. Sensitivity analysis for permanent pacemarker implatation Figure S24 Funnel plot for permanent pacemarker implatation Figure S25 Sensitivity analysis for new‐onset or worsening atrial fibrillation Figure S26 Funnel plot for new‐onset or worsening atrial fibrillation Figure S27 Sensitivity analysis for AKI Figure S28 Funnel plot for AKI Figure S29 Sensitivity analysis for major vascular complications Figure S30 Funnel plot for major vascular complications [file CLC-43-1414-s001.doc]

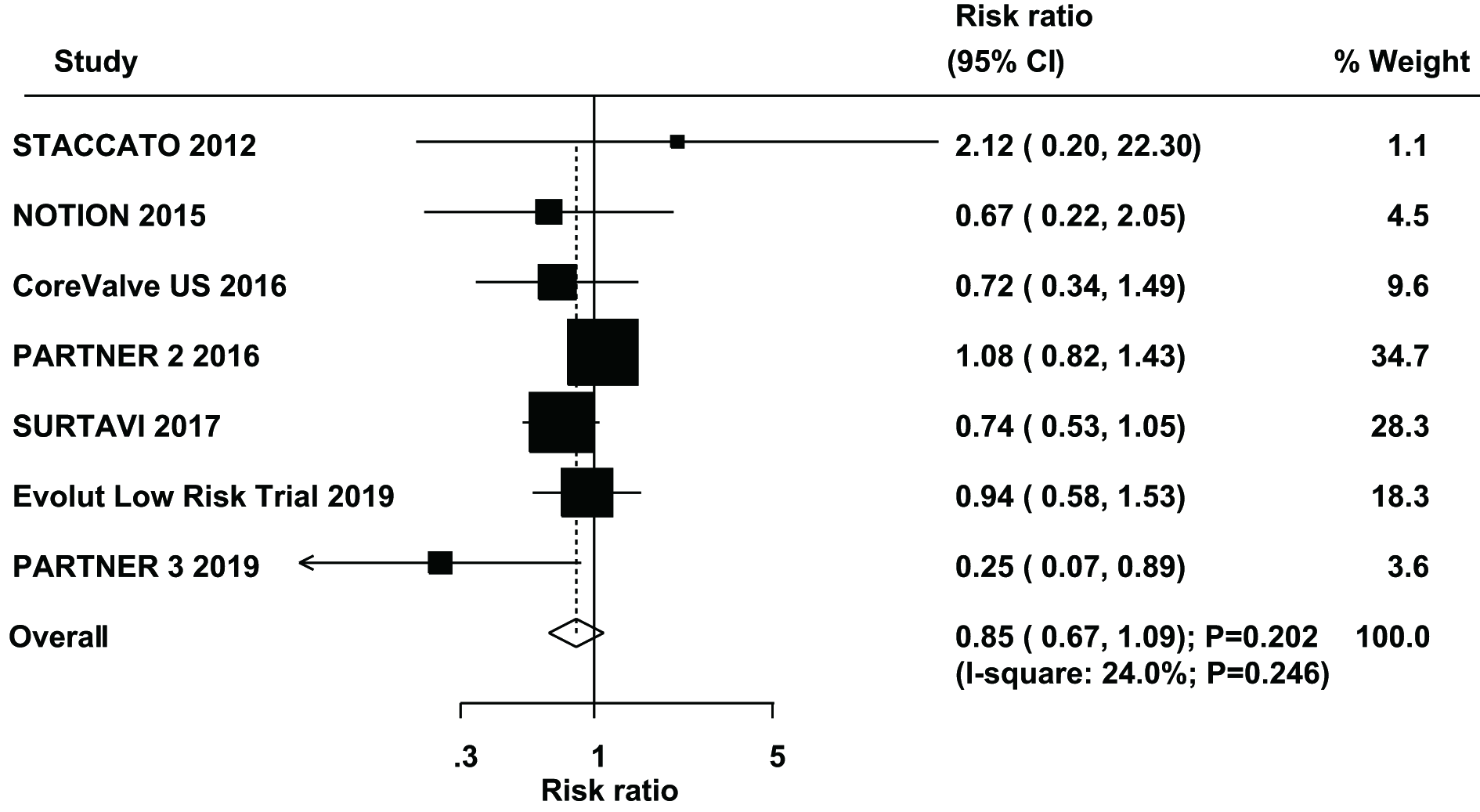


Figure S1. TAVR versus SAVR on the risk of stroke


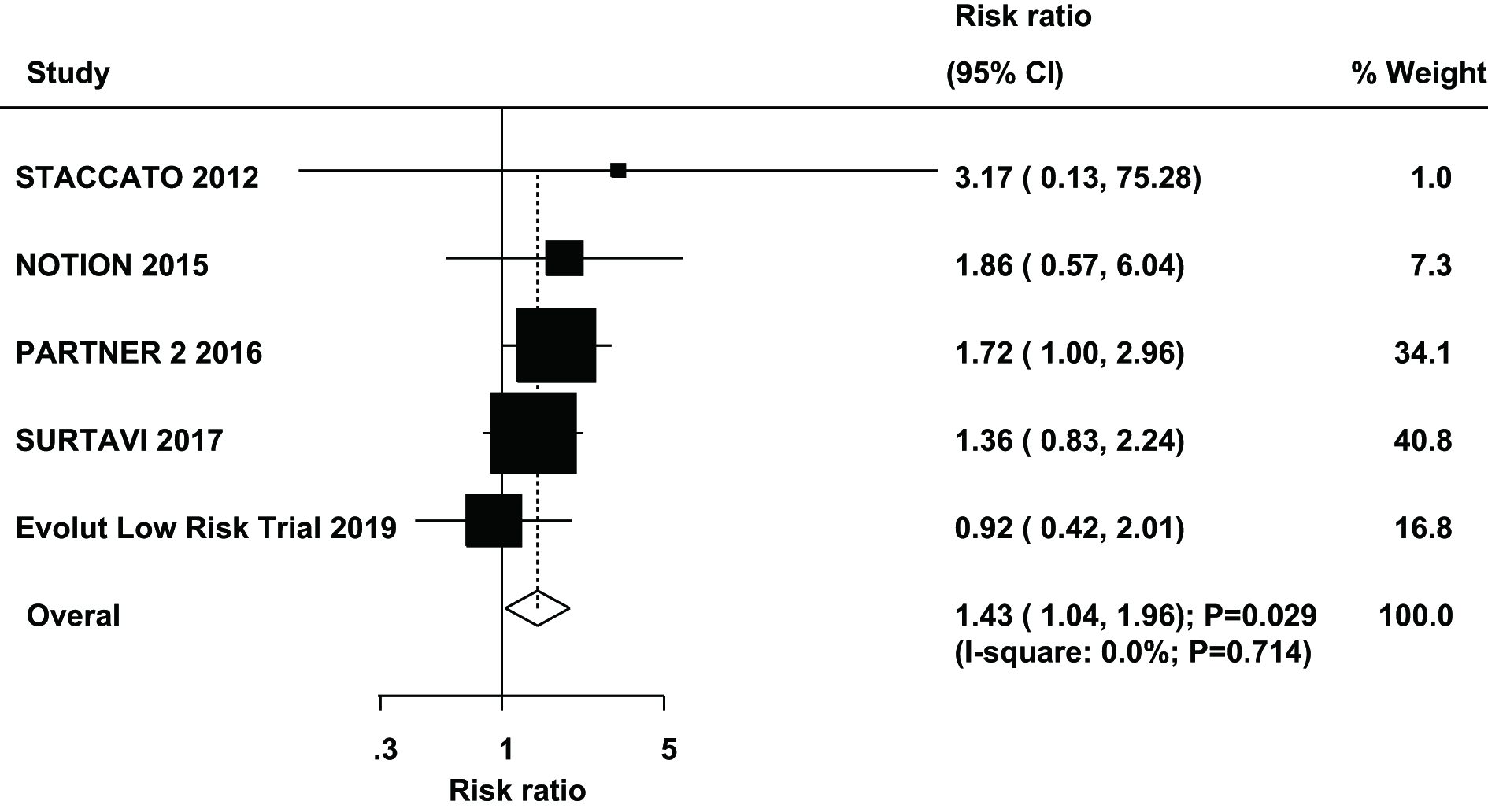


Figure S2. TAVR versus SAVR on the risk of transient ischemic attack


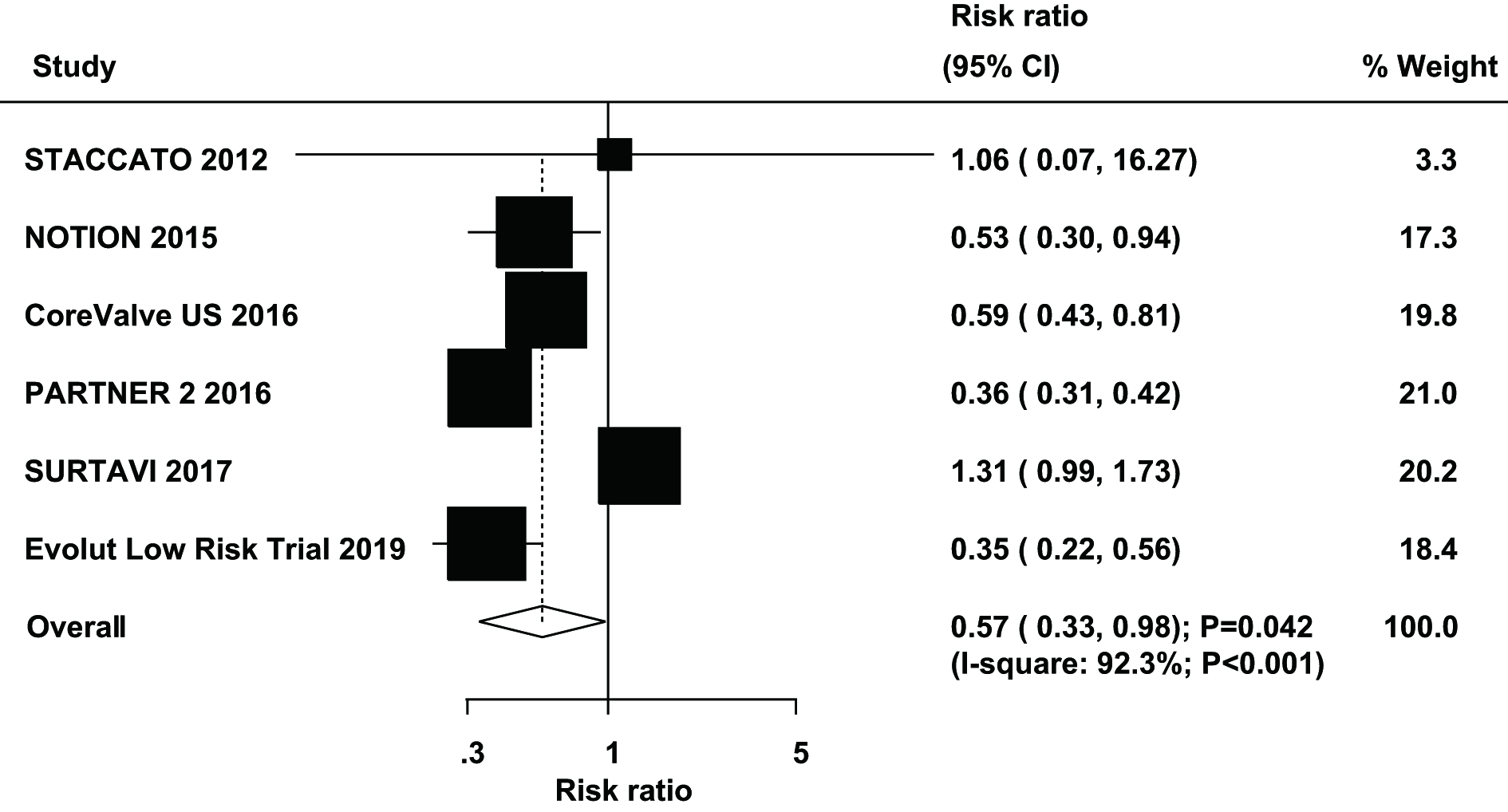


Figure S3. TAVR versus SAVR on the risk of post-procedural bleeding


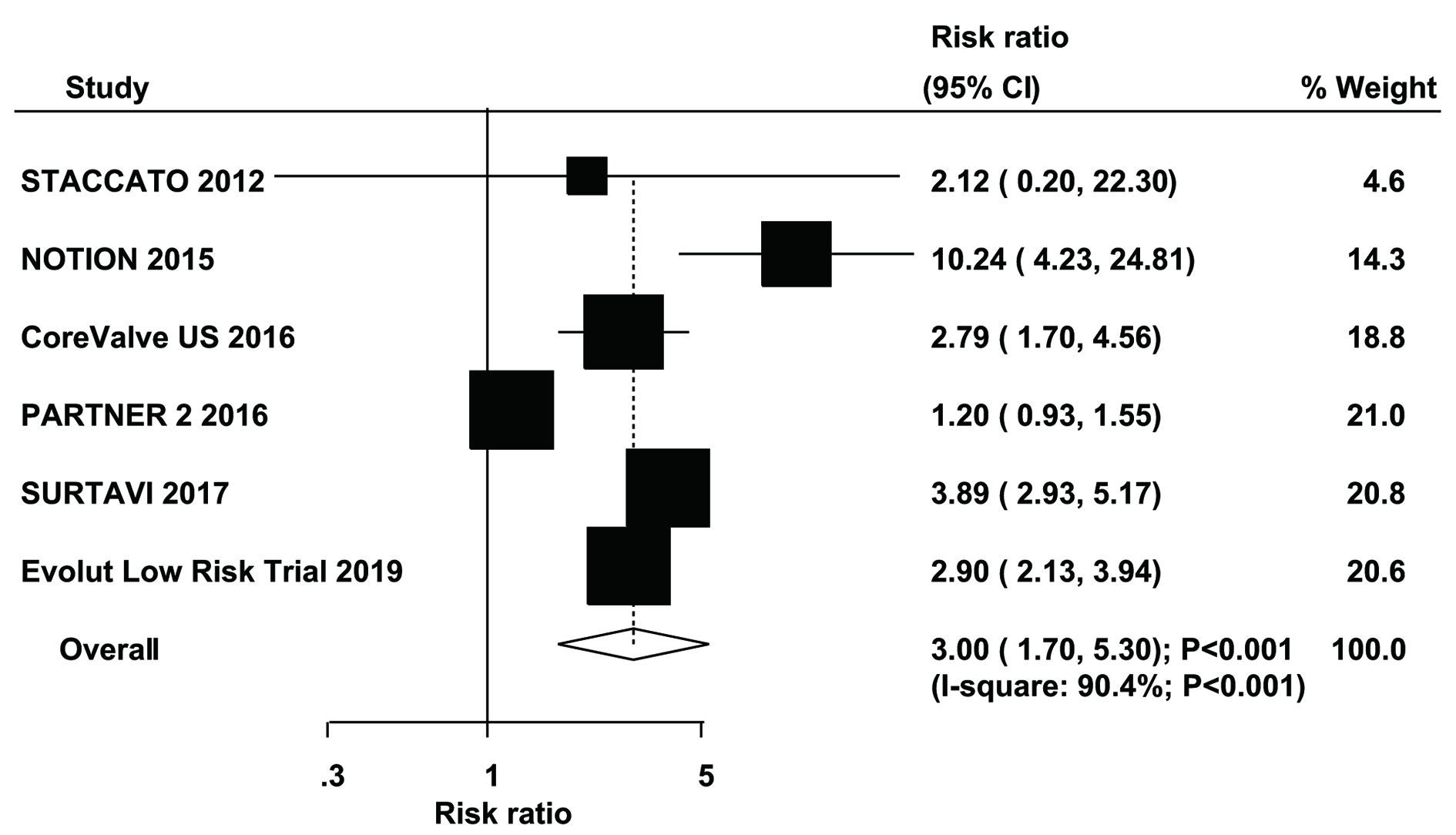


Figure S4. TAVR versus SAVR on the risk of permanent pacemarker implantation


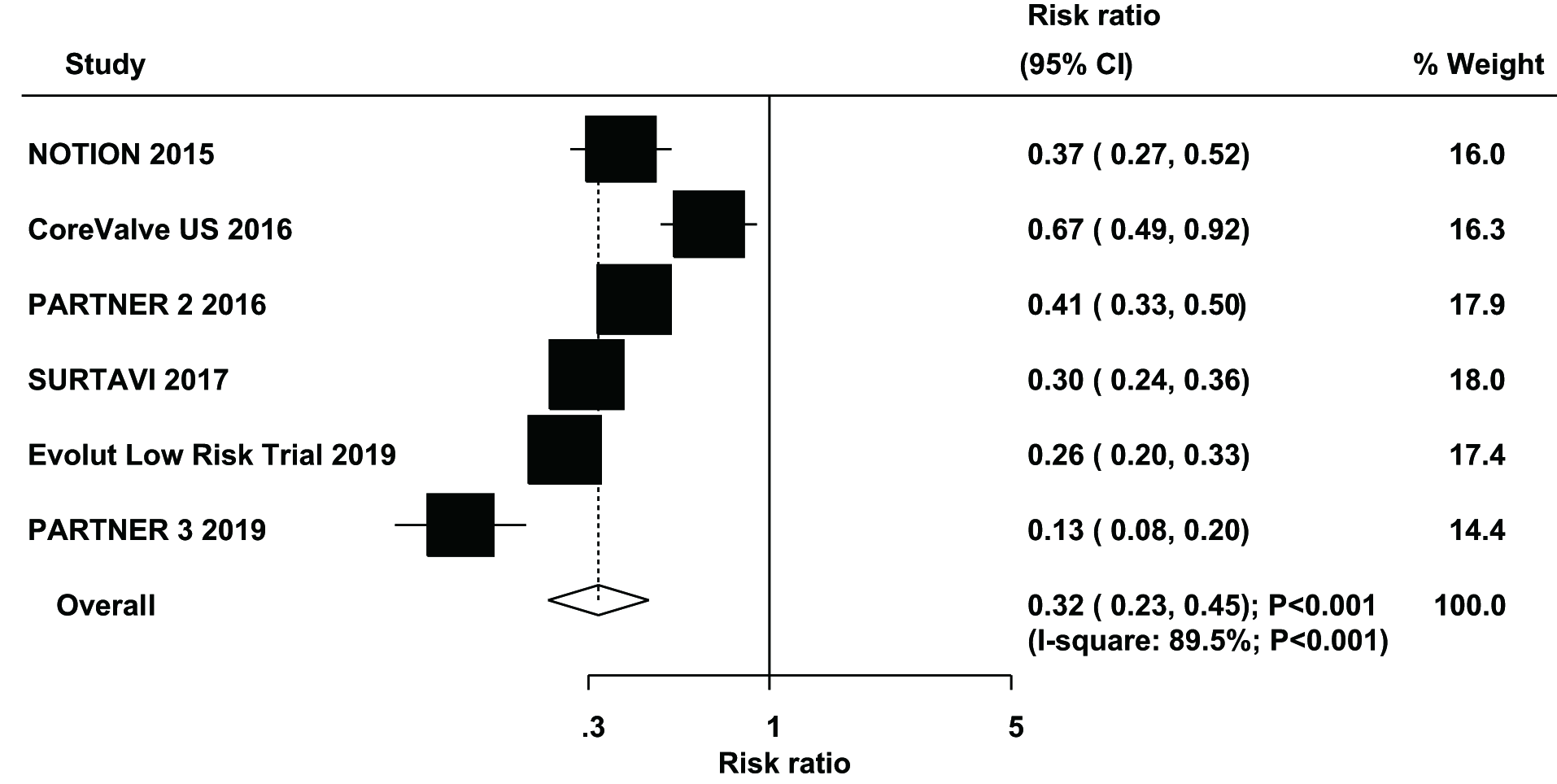


Figure S5. TAVR versus SAVR on the risk of new-onset or worsening atrial fibrillation


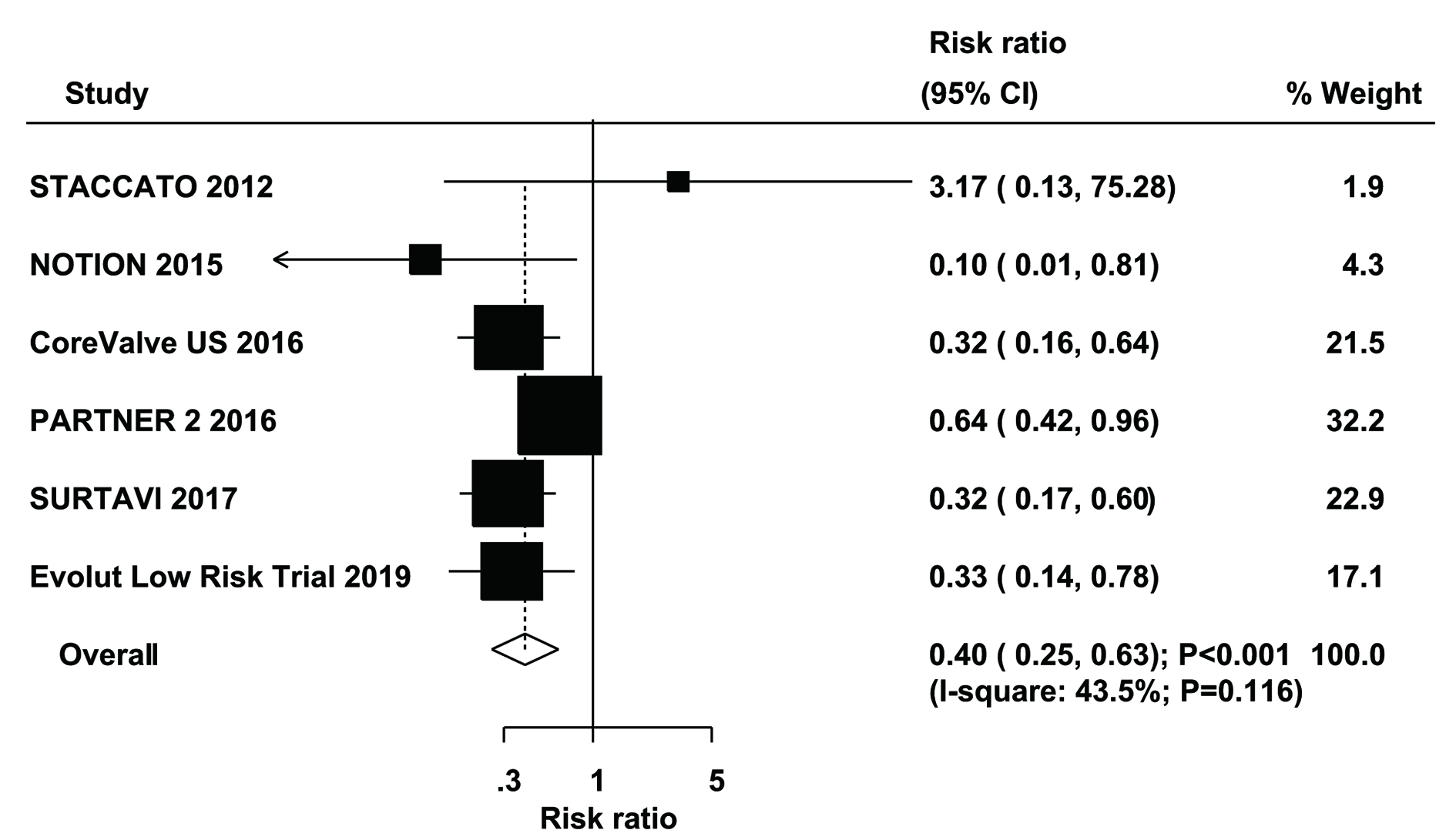


Figure S6. TAVR versus SAVR on the risk of acute kidney injury


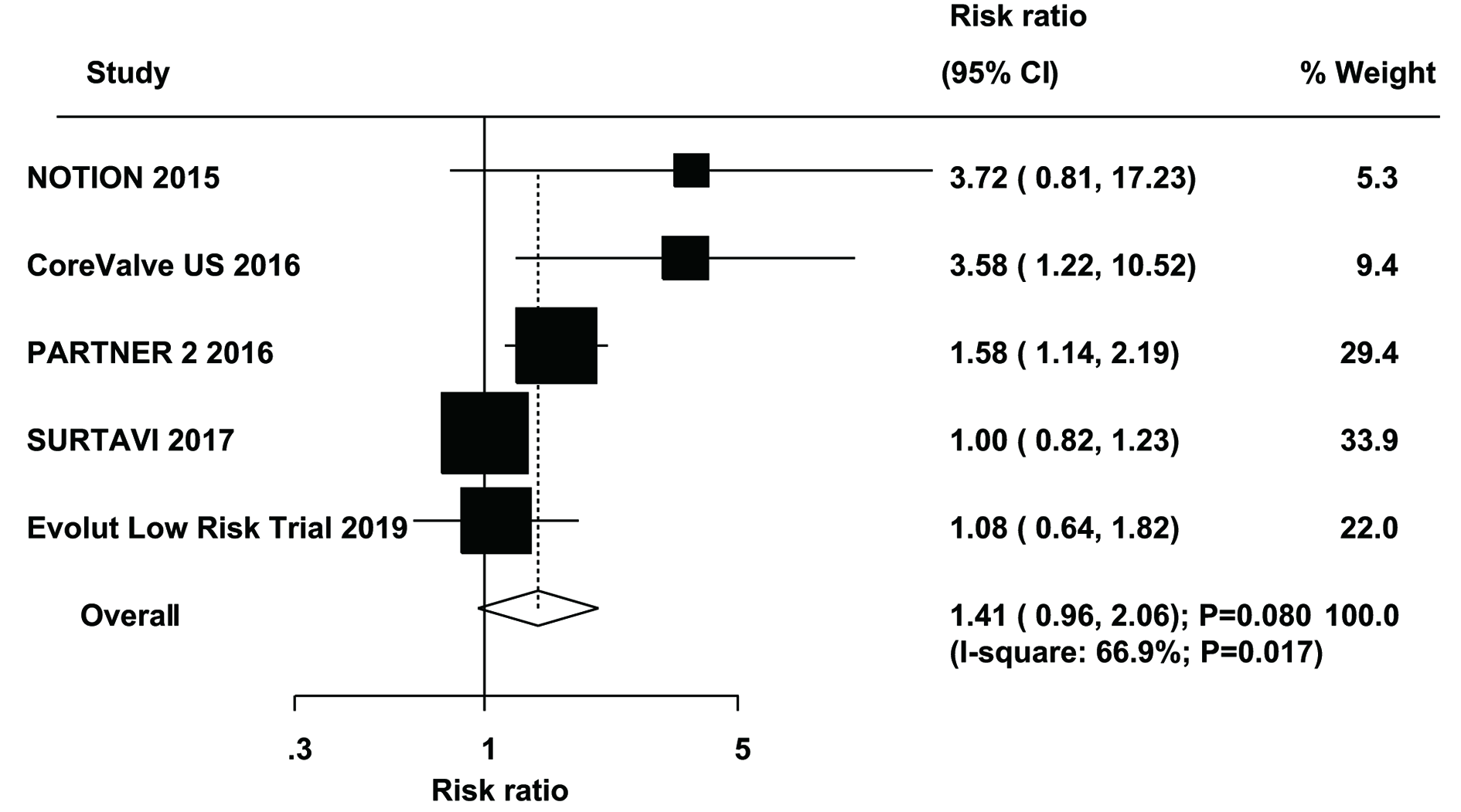


Figure S7. TAVR versus SAVR on the risk of major vascular complications


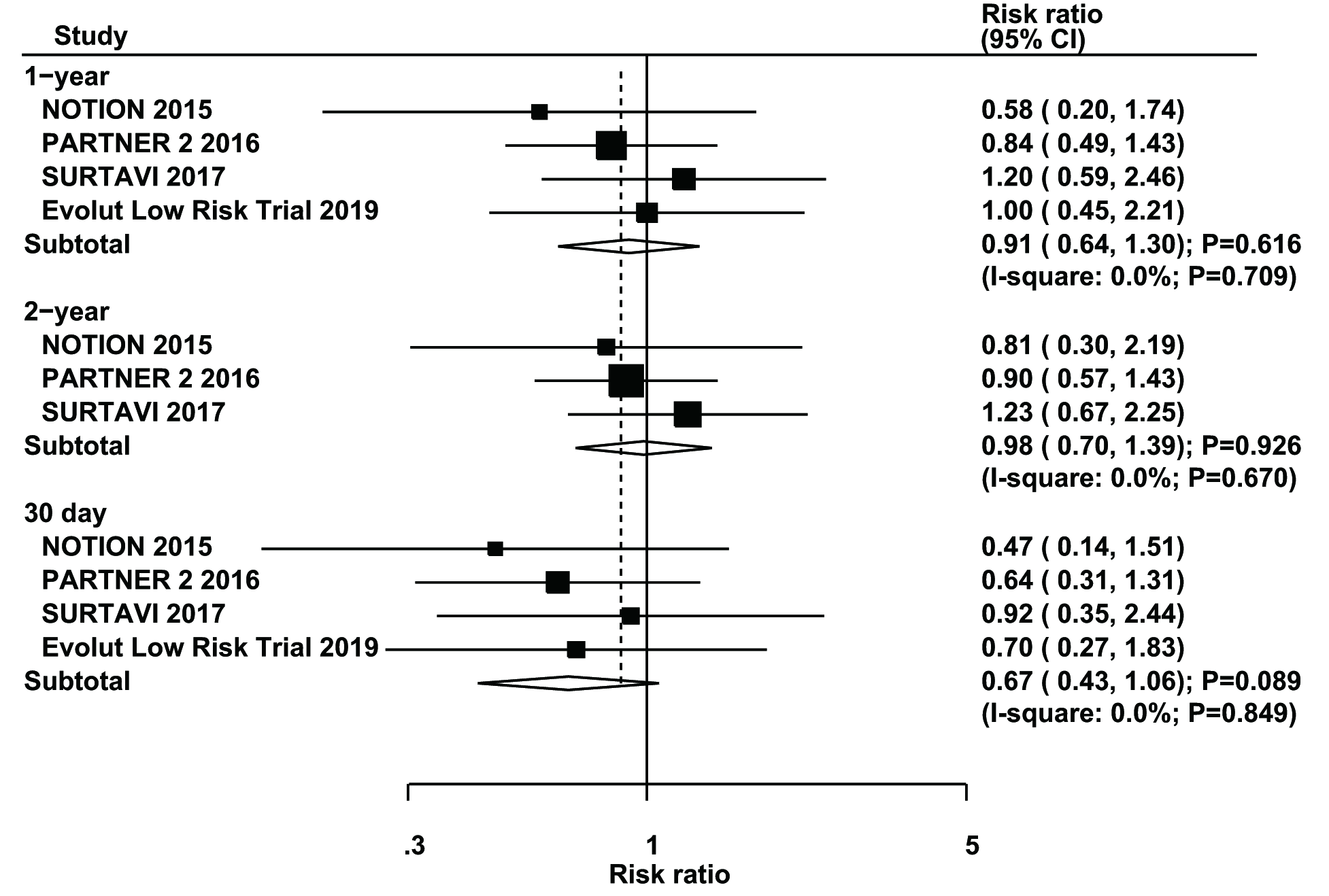


Figure S8. TAVR versus SAVR on the risk of myocardial infarction


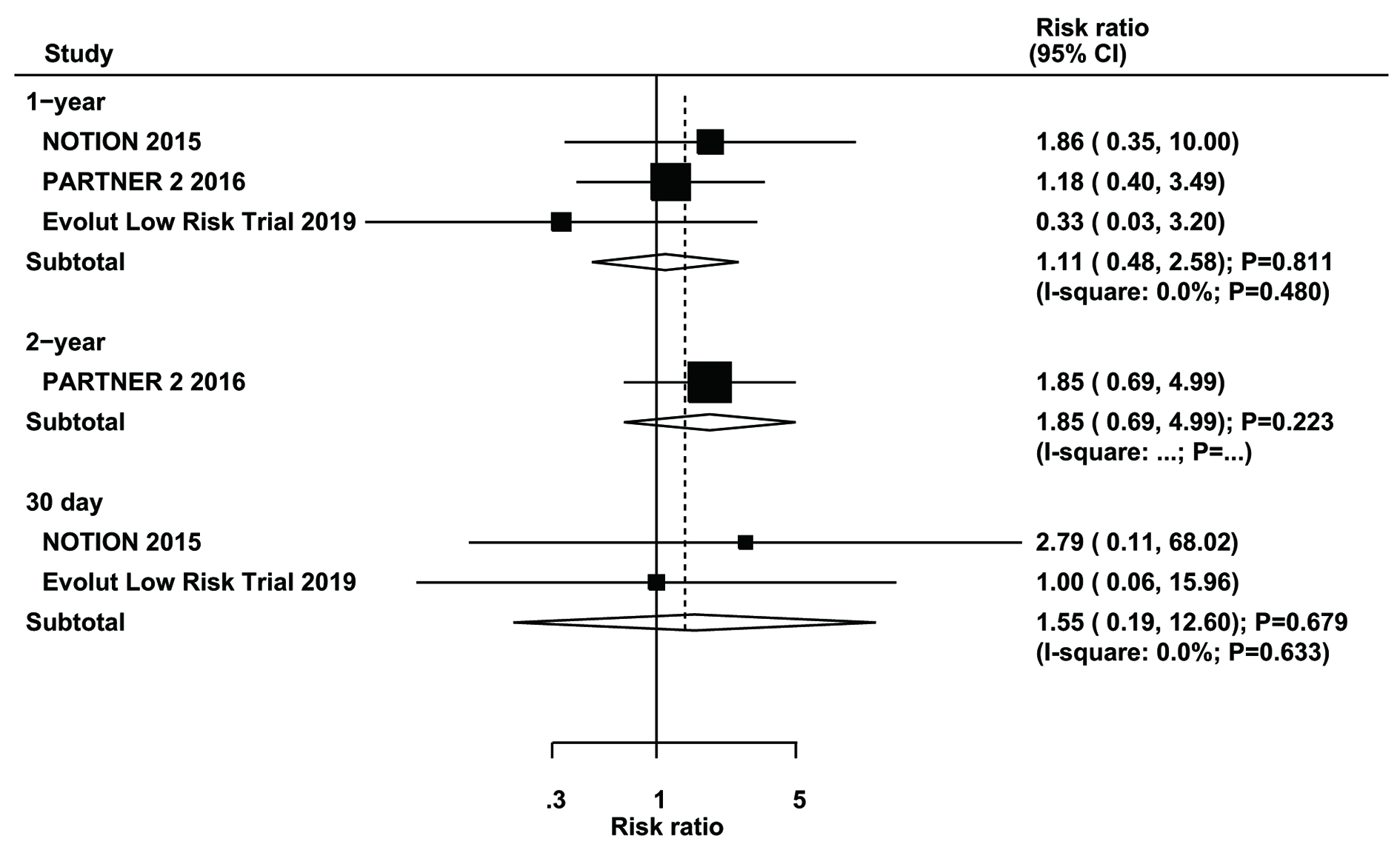


Figure S9. TAVR versus SAVR on the risk of valvular endocarditis


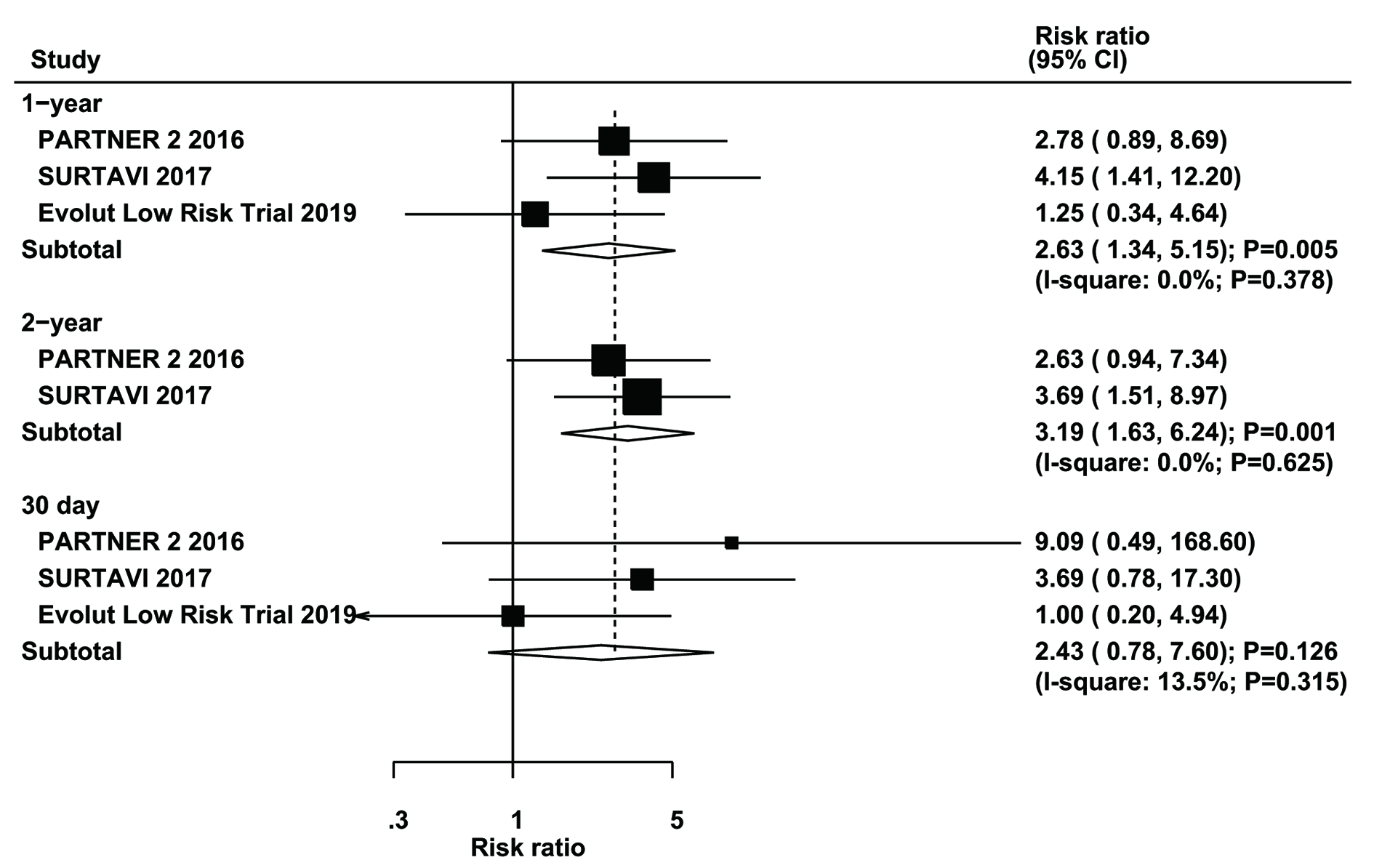


Figure S10. TAVR versus SAVR on the risk of aortic-valve reintervention


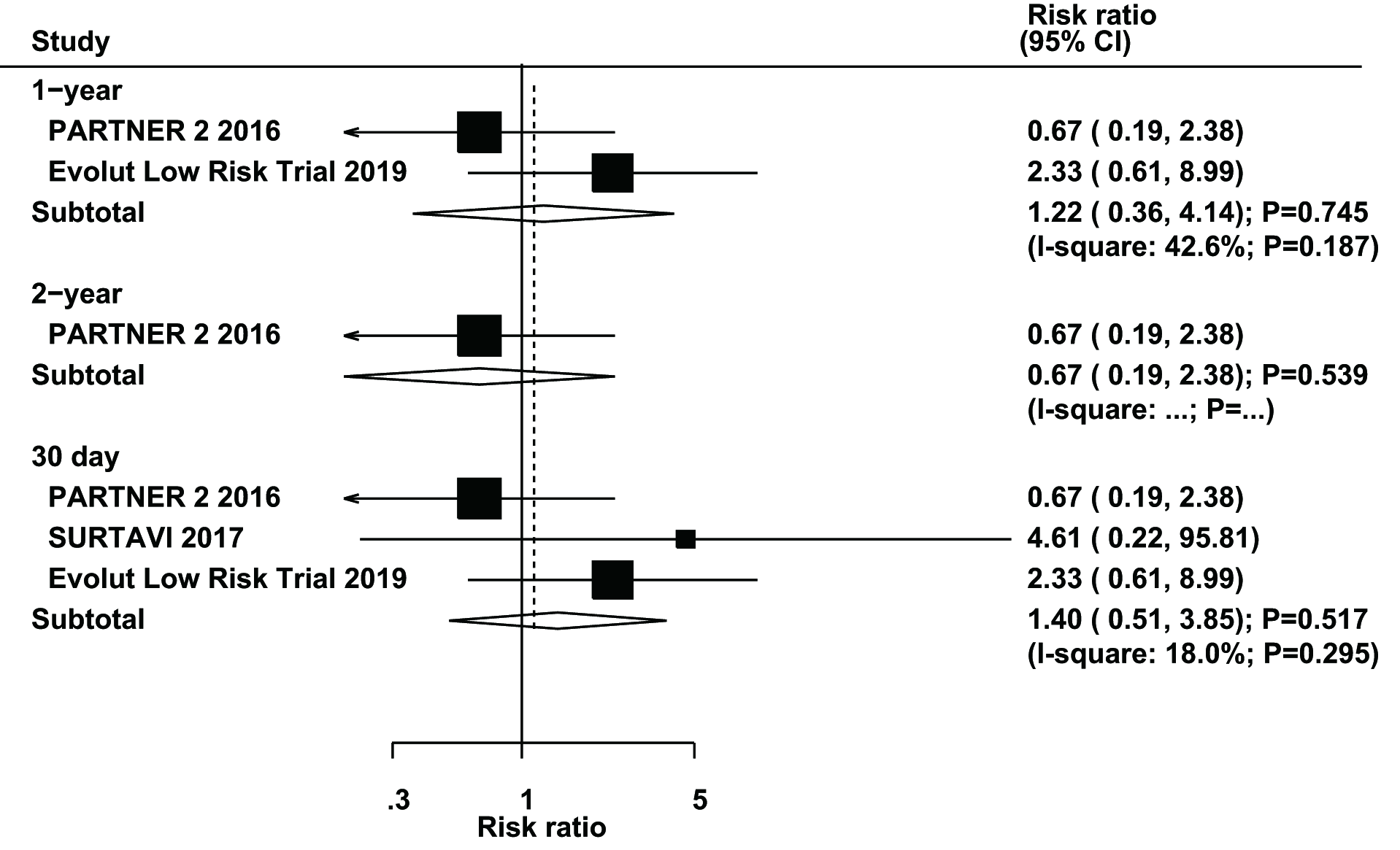


Figure S11. TAVR versus SAVR on the risk of coronary obstruction


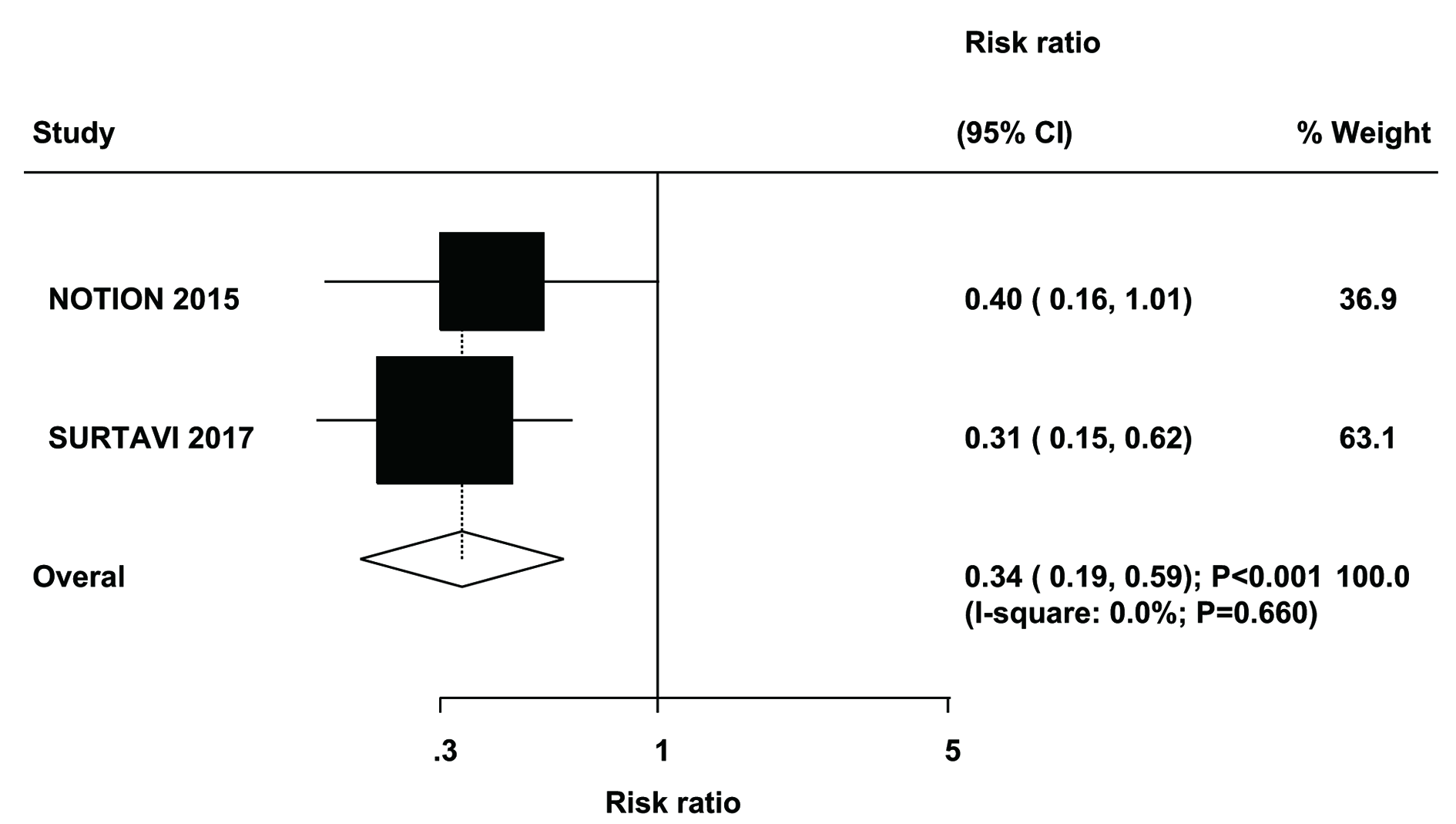


Figure S12. TAVR versus SAVR on the risk of cardiogenic shock.


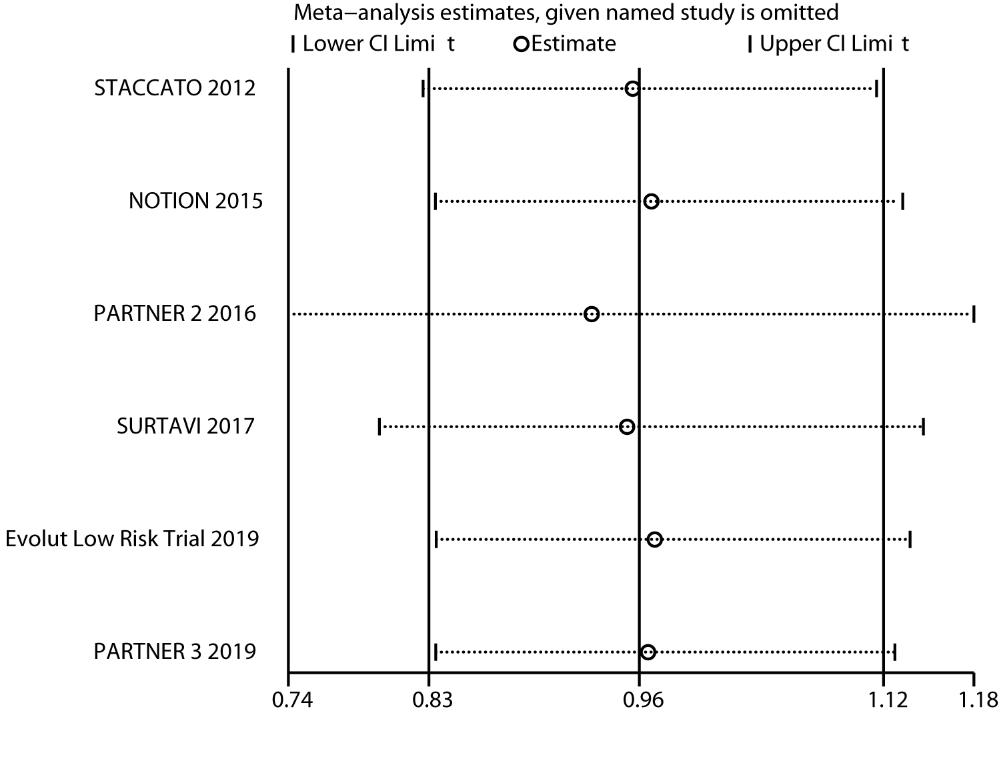


Figure S13 Sensitivity analysis for all-cause mortality


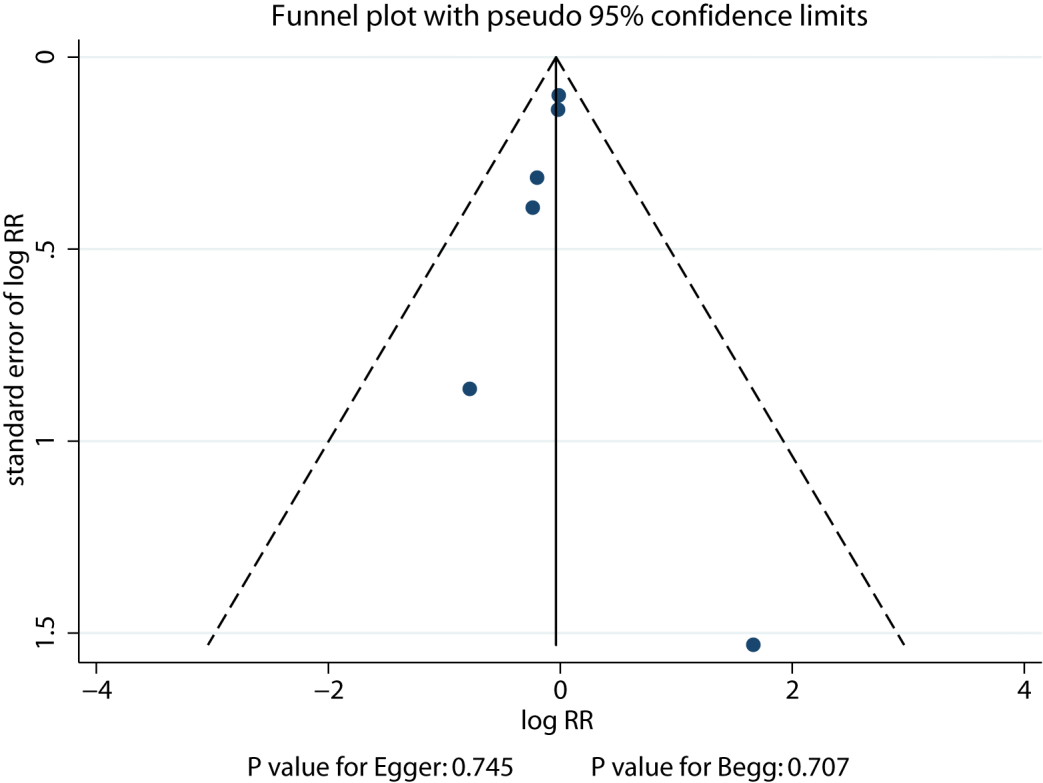


Figure S14 Funnel plot for all-cause mortality


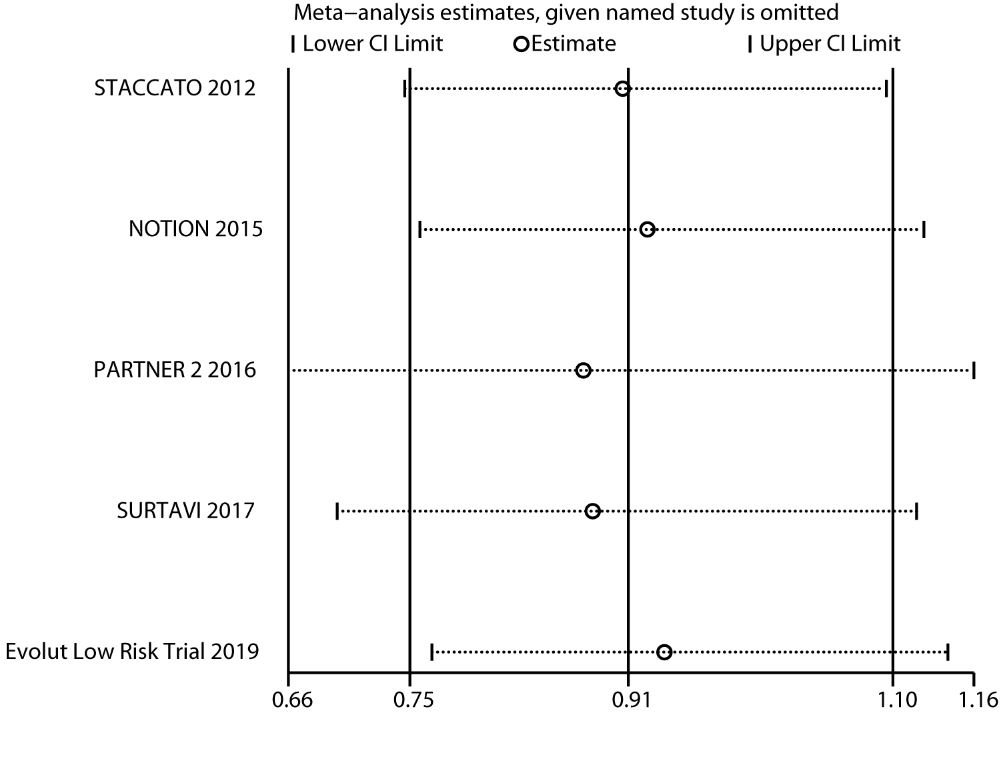


Figure S15 Sensitivity analysis for cardiac death


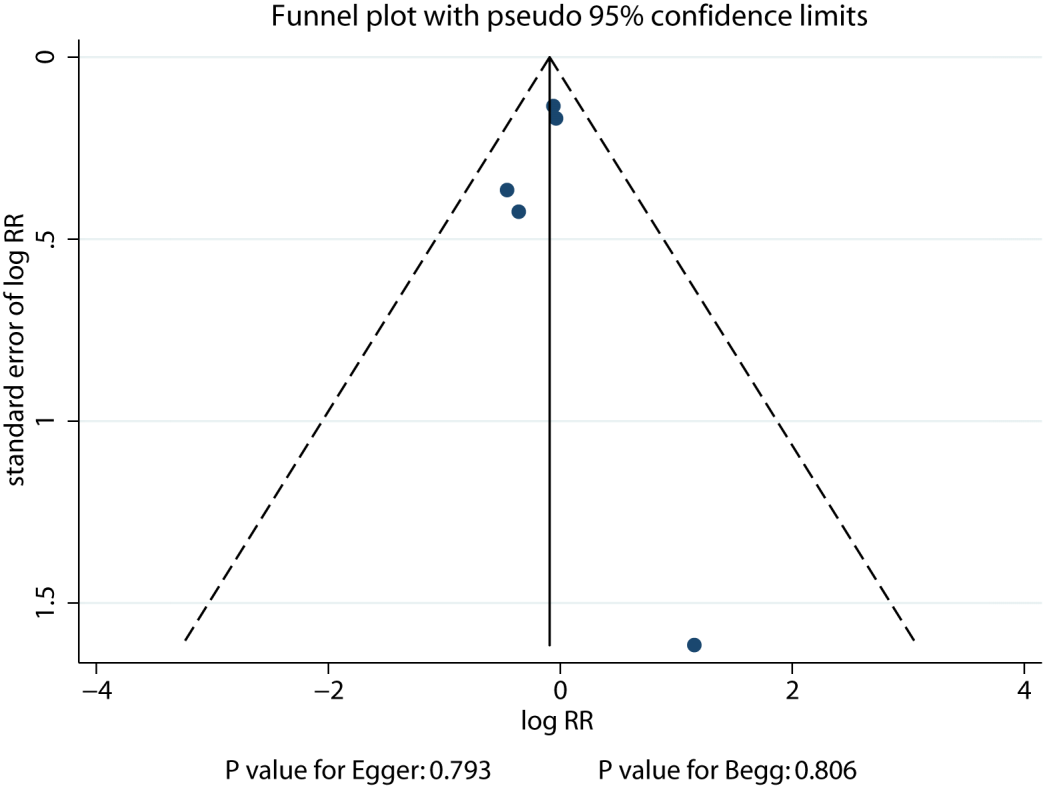


Figure S16 Funnel plot for cardiac death


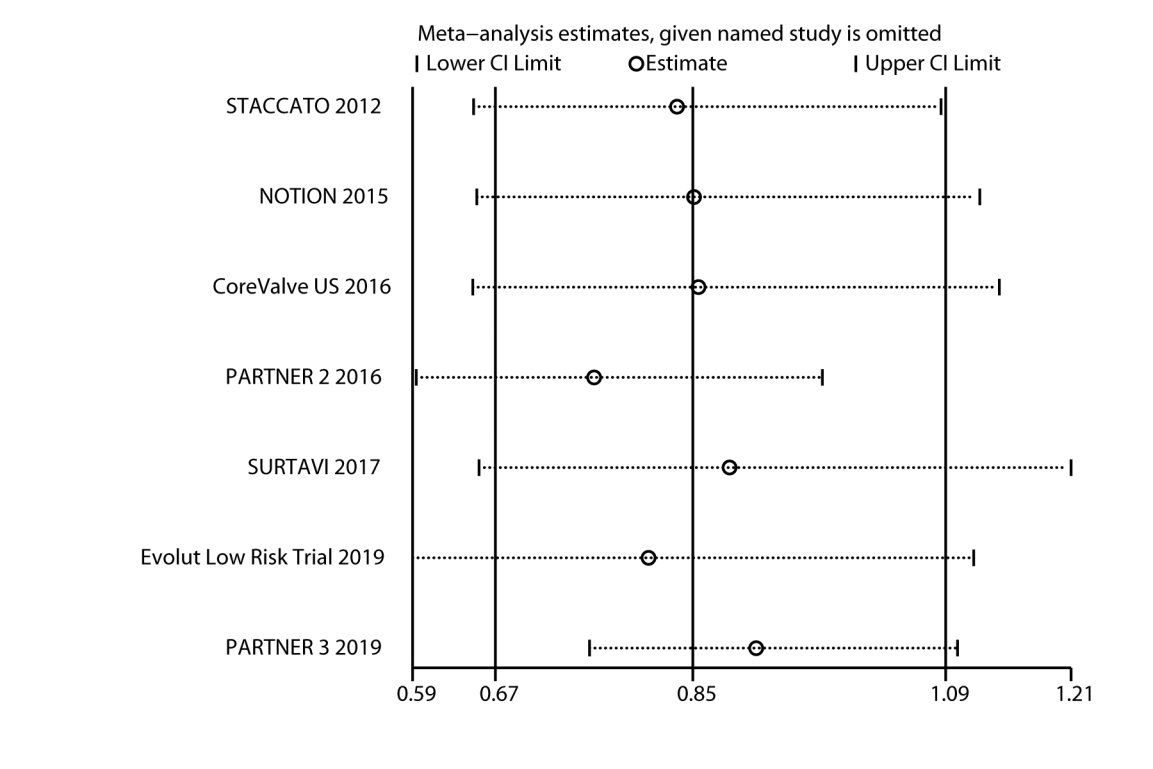


Figure S17 Sensitivity analysis for stroke


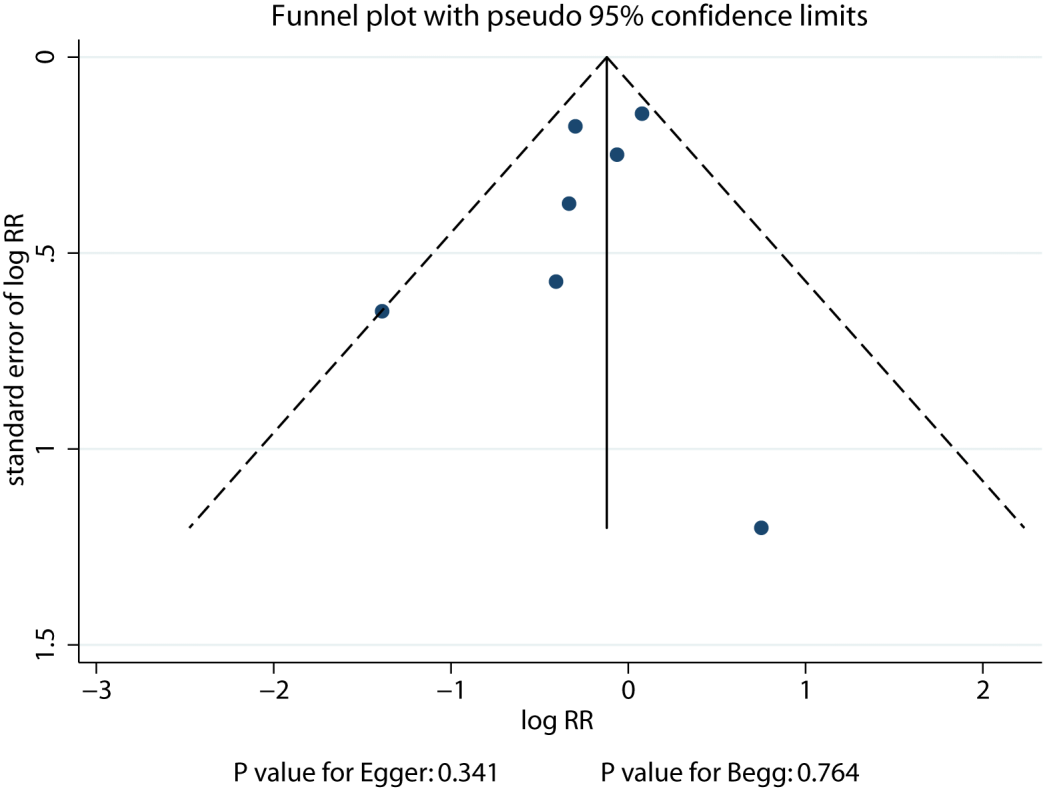


Figure S18 Funnel plot for stroke


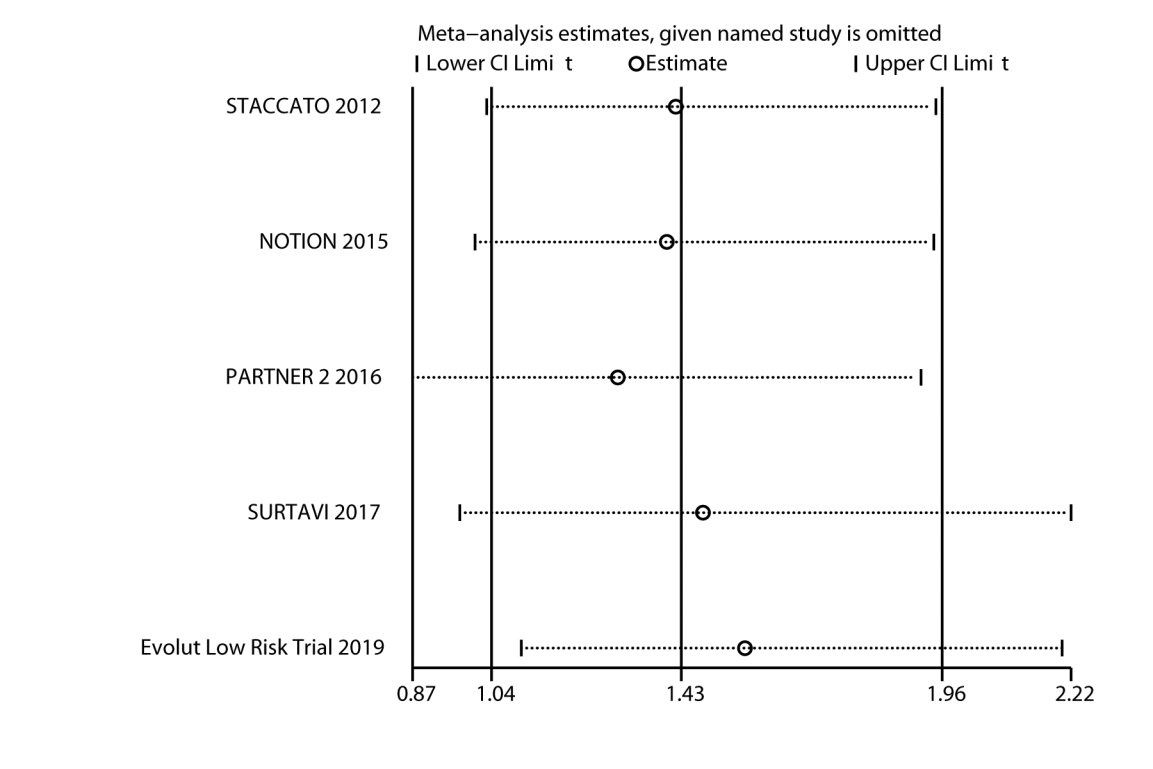


Figure S19 Sensitivity analysis for TIA


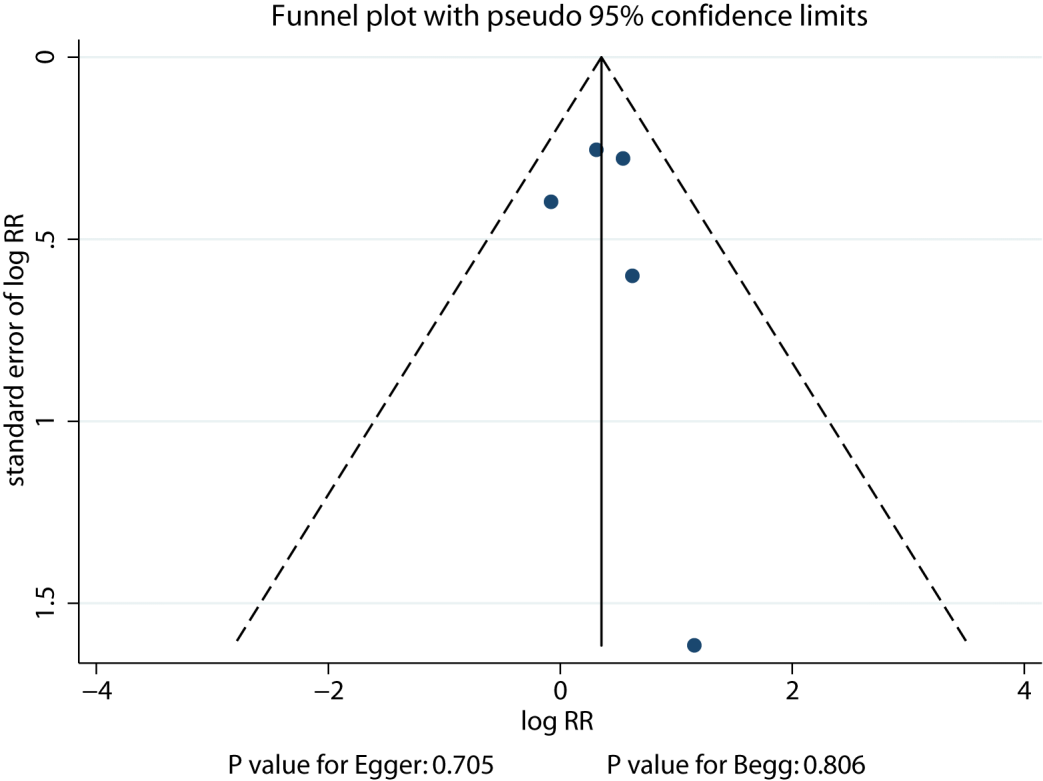


Figure S20 Funnel plot for TIA


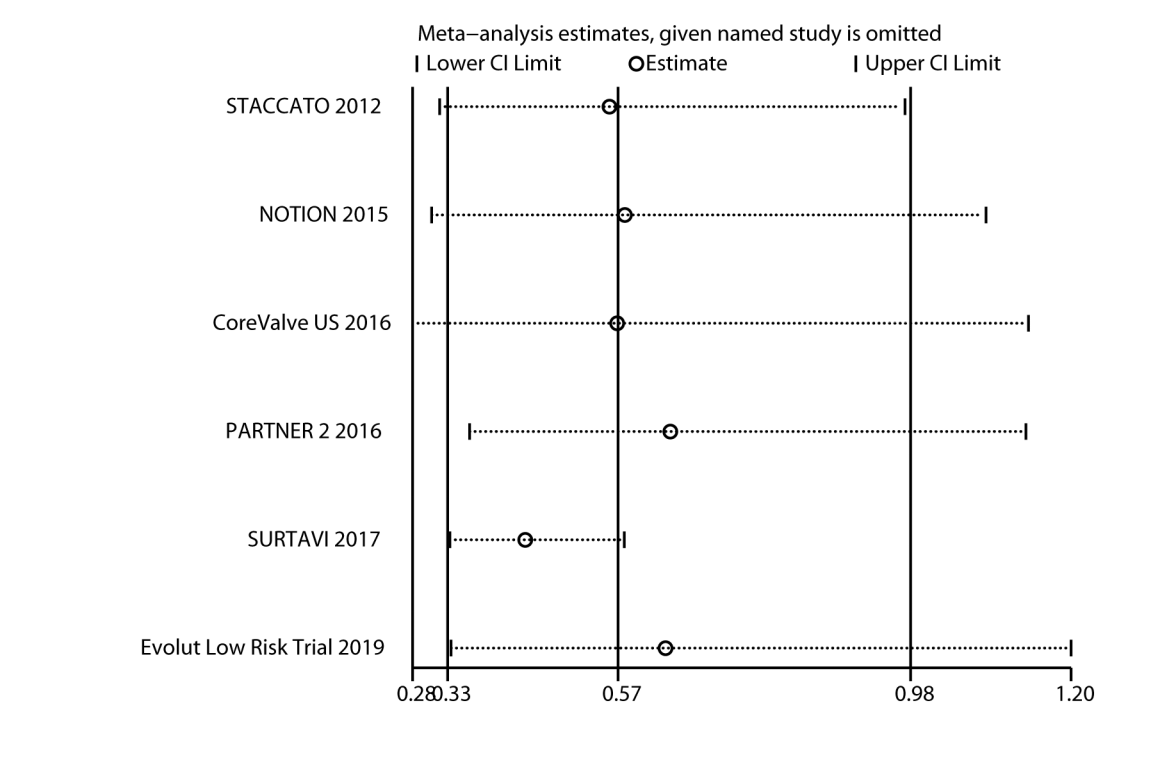


Figure S21 Sensitivity analysis for post-procedural bleeding


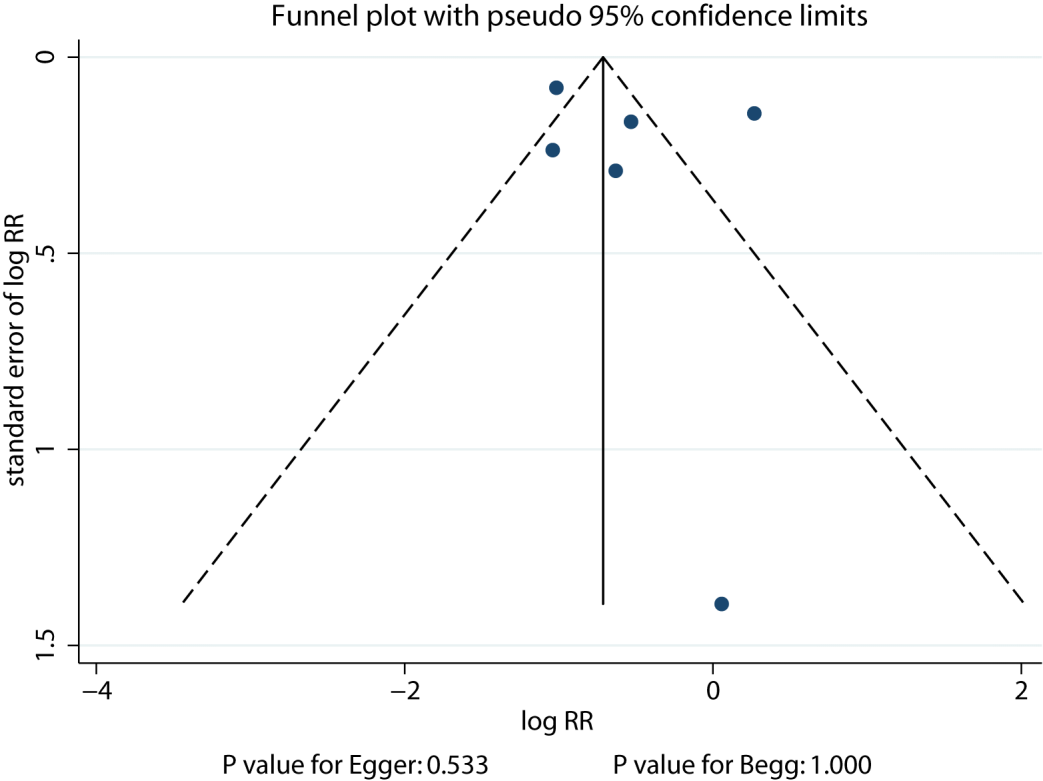


Figure S22 Funnel plot for post-procedural bleeding


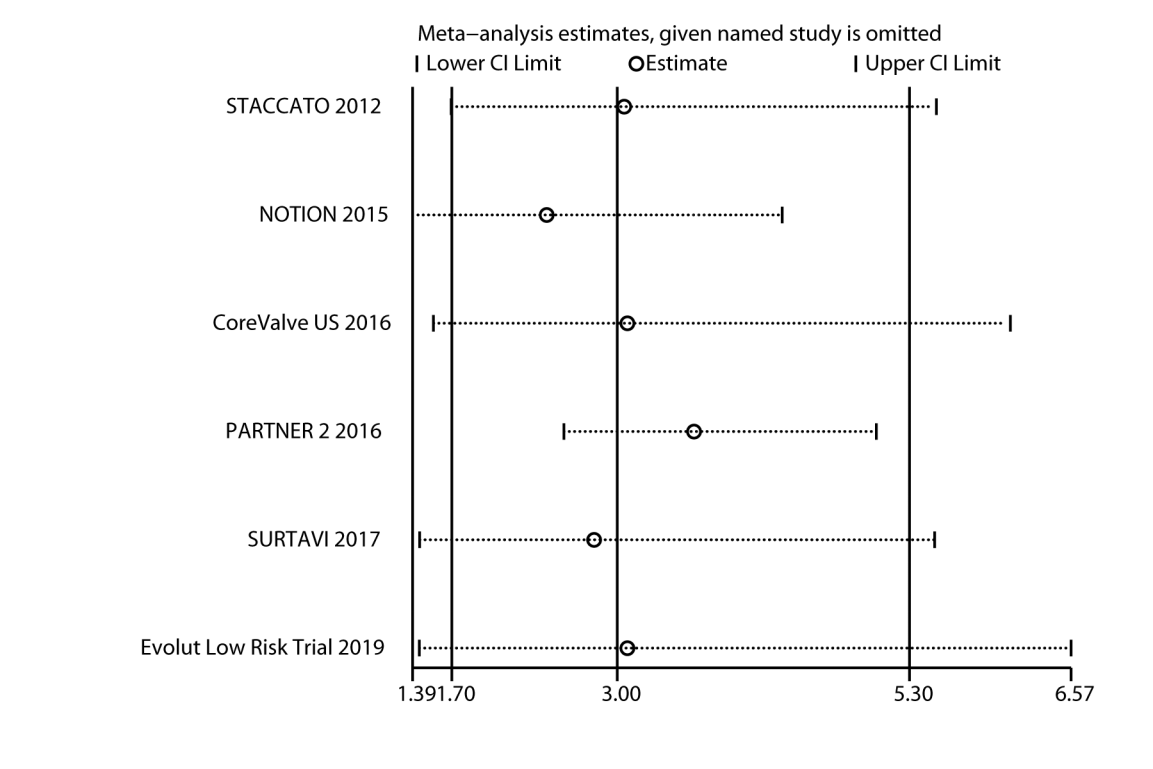


Figure S23. Sensitivity analysis for permanent pacemarker implatation


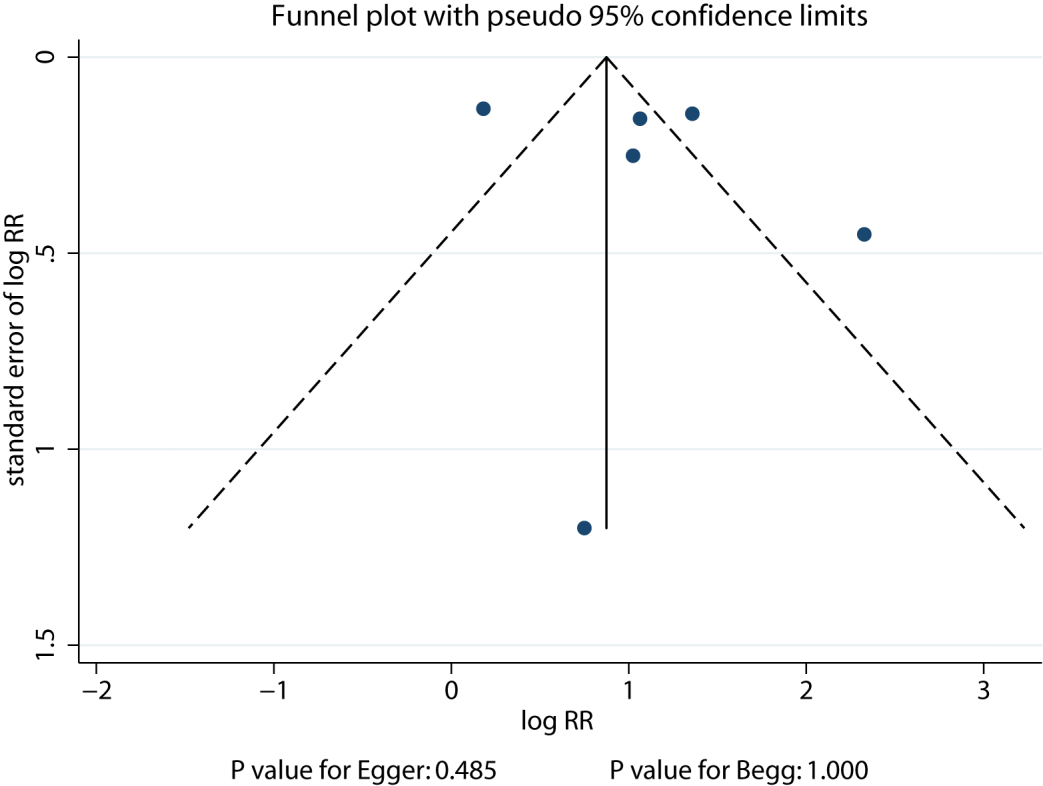


Figure S24 Funnel plot for permanent pacemarker implatation


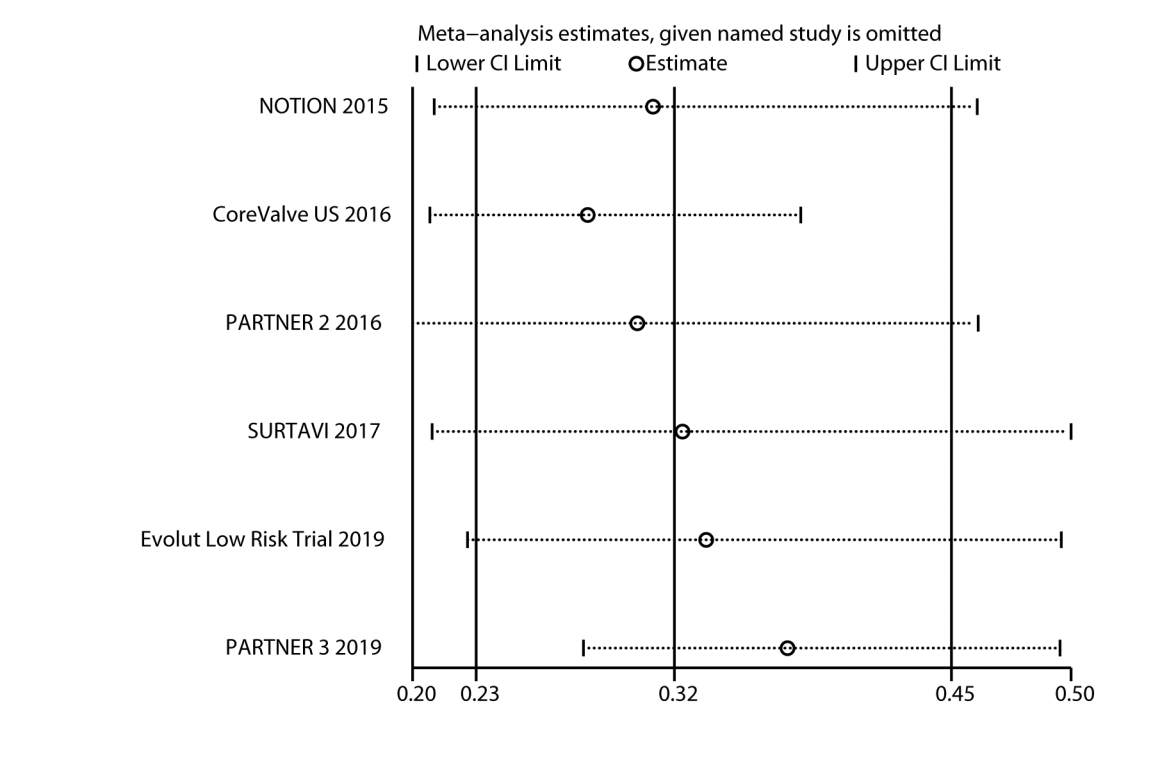


Figure S25 Sensitivity analysis for new-onset or worsening atrial fibrillation


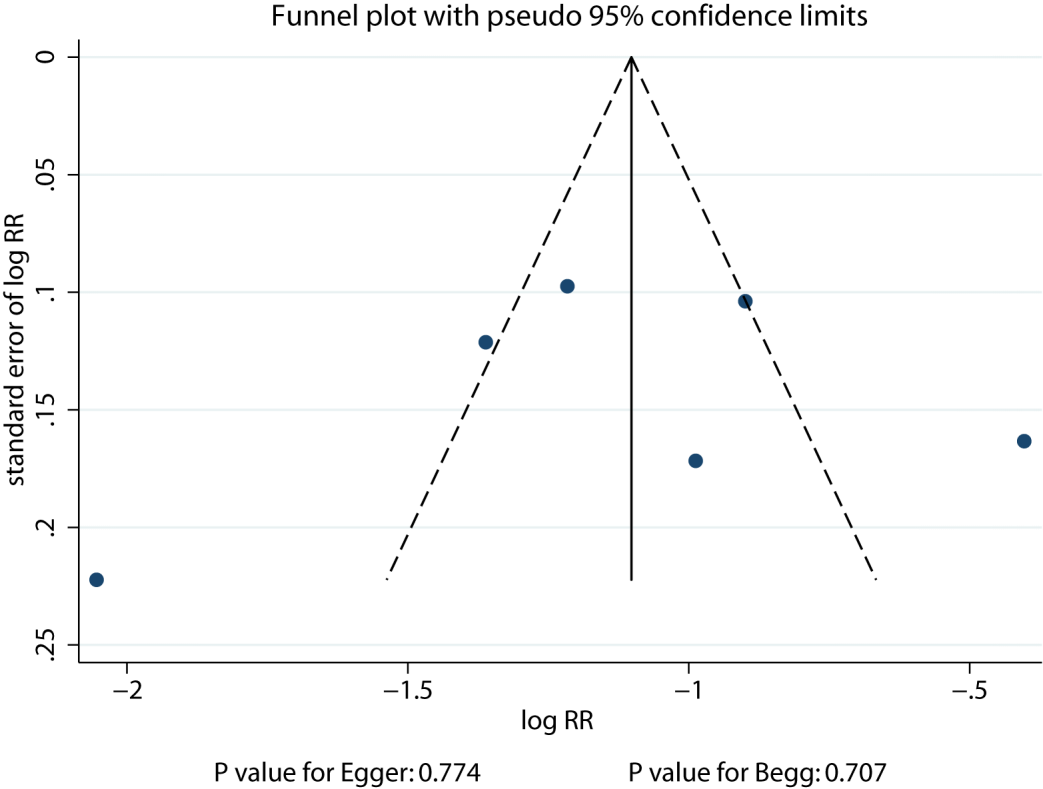


Figure S26 Funnel plot for new-onset or worsening atrial fibrillation


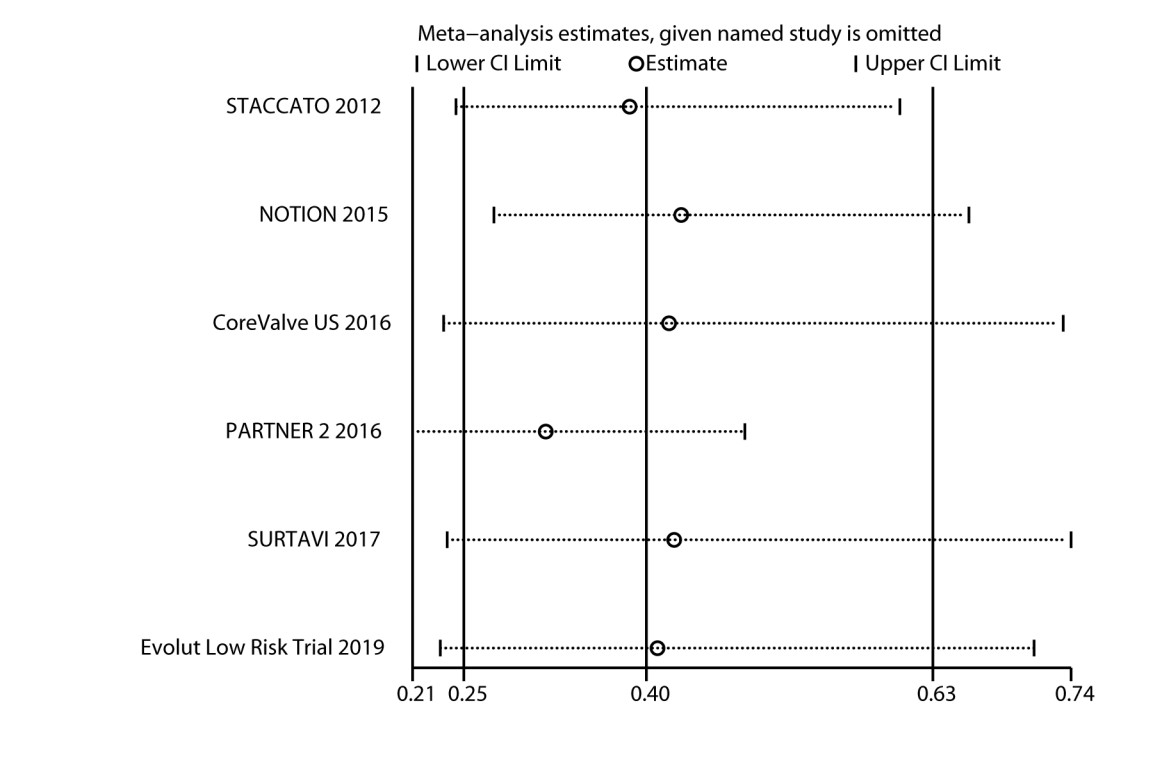


Figure S27 Sensitivity analysis for AKI


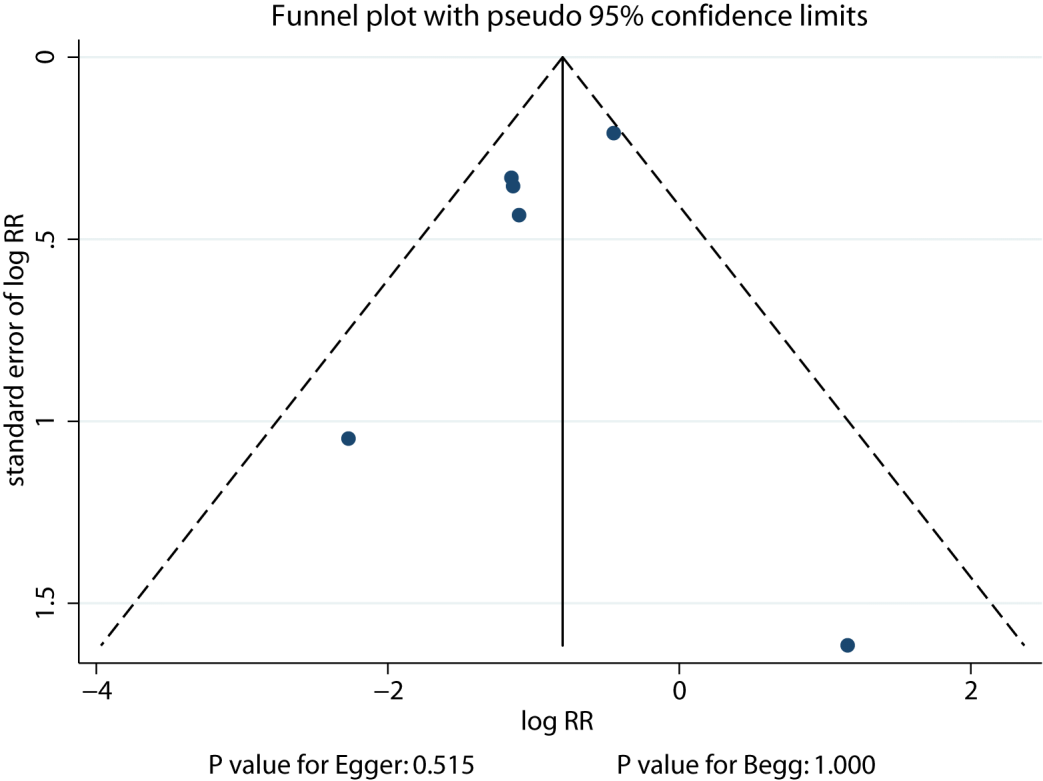


Figure S28 Funnel plot for AKI


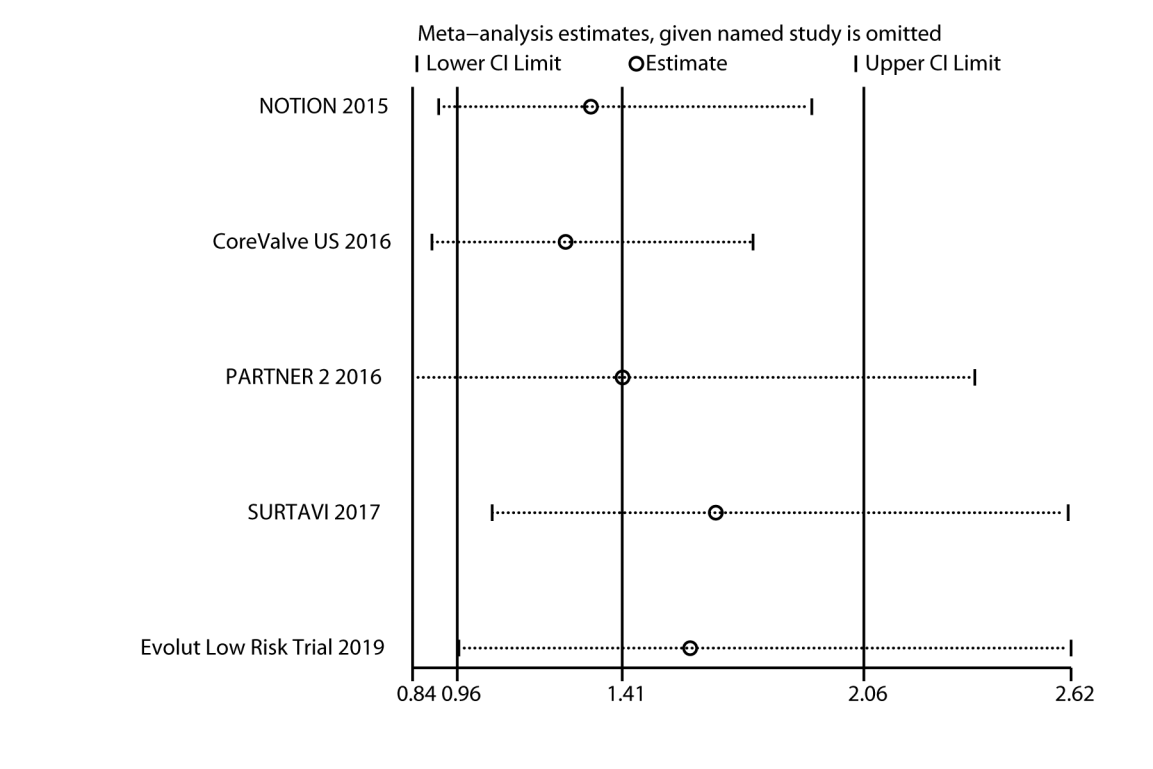


Figure S29 Sensitivity analysis for major vascular complications


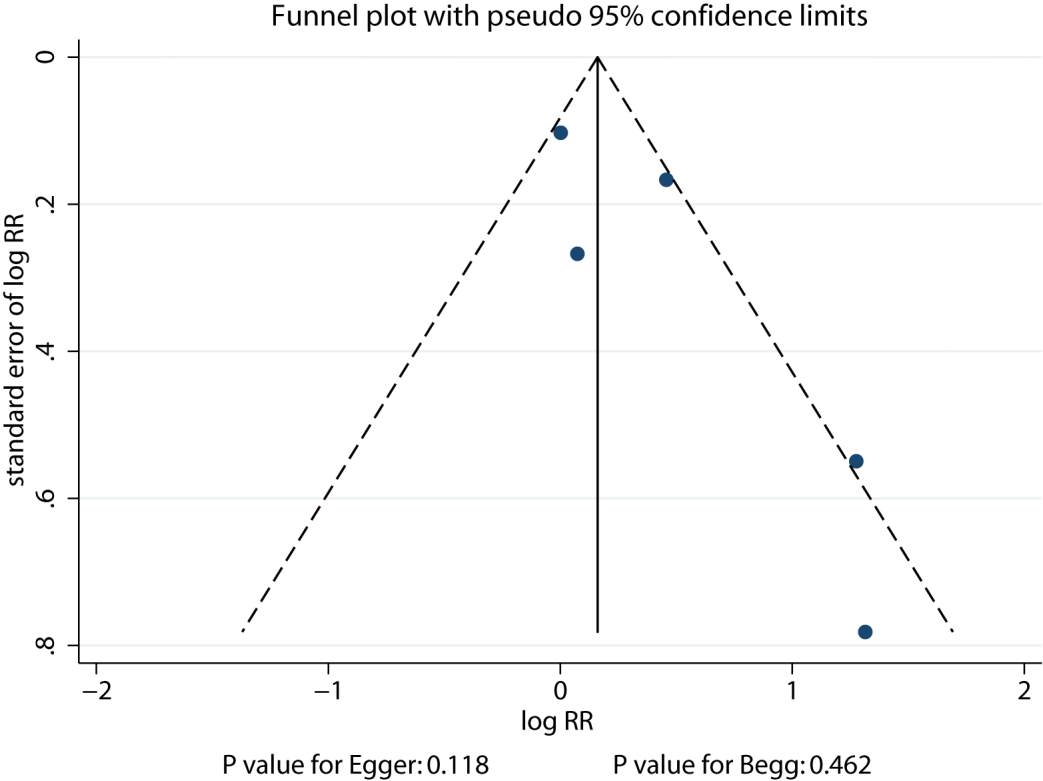


Figure S30 Funnel plot for major vascular complications
