# Supplementary material for: Transcatheter vs surgical aortic valve replacement in low to intermediate surgical risk aortic stenosis patients: A systematic review and meta‐analysis of randomized controlled trials
Source: Clin Cardiol. 2020 Sep 14;43(12):1414–22. doi: 10.1002/clc.23454 (PMC7724228; doi:10.1002/clc.23454)
Supplement: Supplementary file 2 — Table S1 Subgroup analysis for all‐cause mortality Table S2. Subgroup analysis for cardiac death Table S3. Subgroup analysis for stroke Table S4. Subgroup analysis for TIA Table S5. Subgroup analysis for post‐procedural bleeding Table S6. Subgroup analysis for permanent pacemarker implatation Table S7. Subgroup analysis for new‐onset or worsening atrial fibrillation Table S8. Subgroup analysis for acute kidney injury Table S9. Subgroup analysis for major vascular complications Table S10. The results of previous meta‐analyses [file CLC-43-1414-s002.doc]

Table S1. Subgroup analysis for all-cause mortality

| Variable | Group | Number of trials | RR and 95% CI | P value | Heterogeneity (%) | P value for heterogeneity | P value between subgroups |
| --- | --- | --- | --- | --- | --- | --- | --- |
| Sample size | ≥ 1000 | 3 | 0.97 (0.84-1.13) | 0.728 | 0.0 | 0.849 | 0.582 |
|  | < 1000 | 3 | 0.80 (0.40-1.57) | 0.511 | 0.0 | 0.376 |  |
| Mean age | ≥ 80.0 | 2 | 1.14 (0.45-2.89) | 0.780 | 16.7 | 0.273 | 0.645 |
|  | < 80.0 | 4 | 0.92 (0.73-1.16) | 0.504 | 0.0 | 0.759 |  |
| STS score | ≥ 3.5 | 2 | 0.98 (0.84-1.15) | 0.846 | 0.0 | 0.977 | 0.425 |
|  | < 3.5 | 4 | 0.81 (0.51-1.28) | 0.360 | 0.0 | 0.583 |  |
| Percentage of DM (%) | ≥ 30.0 | 4 | 0.97 (0.83-1.13) | 0.669 | 0.0 | 0.780 | 0.883 |
|  | < 30.0 | 2 | 1.16 (0.25-5.29) | 0.847 | 32.4 | 0.224 |  |
| Prior stroke (%) | ≥ 10.0 | 3 | 0.96 (0.80-1.15) | 0.644 | 0.0 | 0.745 | 0.920 |
|  | < 10.0 | 3 | 0.98 (0.75-1.27) | 0.857 | 0.0 | 0.370 |  |
| Prior PVD (%) | ≥ 10.0 | 2 | 0.98 (0.84-1.15) | 0.846 | 0.0 | 0.977 | 0.425 |
|  | < 10.0 | 4 | 0.81 (0.51-1.28) | 0.360 | 0.0 | 0.583 |  |
| Prior MI (%) | ≥ 10.0 | 2 | 0.98 (0.84-1.15) | 0.846 | 0.0 | 0.977 | 0.425 |
|  | < 10.0 | 4 | 0.81 (0.51-1.28) | 0.360 | 0.0 | 0.583 |  |
| Prior COPD (%) | ≥ 10.0 | 3 | 0.96 (0.80-1.15) | 0.644 | 0.0 | 0.745 | 0.989 |
|  | < 10.0 | 2 | 1.13 (0.11-11.70) | 0.919 | 49.4 | 0.160 |  |
|  | Not reported | 1 | 0.98 (0.75-1.28) | 0.891 | - | - |  |
| Percentage of NYHA III-IV | ≥ 50.0 | 2 | 0.98 (0.84-1.15) | 0.846 | 0.0 | 0.977 | 0.425 |
|  | < 50.0 | 4 | 0.81 (0.51-1.28) | 0.360 | 0.0 | 0.583 |  |
| Valve type | Balloon-expanding | 3 | 0.98 (0.81-1.19) | 0.863 | 0.0 | 0.369 | 0.756 |
|  | Self-expanding | 3 | 0.94 (0.74-1.18) | 0.583 | 0.0 | 0.779 |  |
| Follow-up duration | 30 days | 6 | 0.91 (0.64-1.29) | 0.591 | 4.7 | 0.386 | 0.944 |
|  | 1 year | 4 | 0.96 (0.80-1.16) | 0.701 | 0.0 | 0.785 |  |
|  | 2 years | 3 | 0.98 (0.84-1.14) | 0.754 | 0.0 | 0.856 |  |
| Study quality | High | 4 | 0.97 (0.83-1.13) | 0.669 | 0.0 | 0.780 | 0.883 |
|  | Low | 2 | 1.16 (0.25-5.29) | 0.847 | 32.4 | 0.224 |  |

Table S2. Subgroup analysis for cardiac death

| Variable | Group | Number of trials | RR and 95% CI | P value | Heterogeneity (%) | P value for heterogeneity | P value between subgroups |
| --- | --- | --- | --- | --- | --- | --- | --- |
| Sample size | ≥ 1000 | 3 | 0.92 (0.76-1.12) | 0.417 | 0.0 | 0.556 | 0.675 |
|  | < 1000 | 2 | 0.77 (0.34-1.72) | 0.524 | 0.0 | 0.364 |  |
| Mean age | ≥ 80.0 | 2 | 0.95 (0.73-1.23) | 0.700 | 0.0 | 0.454 | 0.659 |
|  | < 80.0 | 3 | 0.87 (0.66-1.15) | 0.334 | 0.0 | 0.492 |  |
| STS score | ≥ 3.5 | 2 | 0.95 (0.77-1.17) | 0.629 | 0.0 | 0.912 | 0.271 |
|  | < 3.5 | 3 | 0.69 (0.40-1.18) | 0.173 | 0.0 | 0.621 |  |
| Percentage of DM (%) | ≥ 30.0 | 3 | 0.92 (0.76-1.12) | 0.417 | 0.0 | 0.556 | 0.675 |
|  | < 30.0 | 2 | 0.77 (0.34-1.72) | 0.524 | 0.0 | 0.364 |  |
| Prior stroke (%) | ≥ 10.0 | 3 | 0.88 (0.69-1.11) | 0.290 | 0.0 | 0.501 | 0.614 |
|  | < 10.0 | 2 | 0.98 (0.70-1.35) | 0.888 | 0.0 | 0.464 |  |
| Prior PVD (%) | ≥ 10.0 | 2 | 0.95 (0.77-1.17) | 0.629 | 0.0 | 0.912 | 0.271 |
|  | < 10.0 | 3 | 0.69 (0.40-1.18) | 0.173 | 0.0 | 0.621 |  |
| Prior MI (%) | ≥ 10.0 | 2 | 0.95 (0.77-1.17) | 0.629 | 0.0 | 0.912 | 0.271 |
|  | < 10.0 | 3 | 0.69 (0.40-1.18) | 0.173 | 0.0 | 0.621 |  |
| Prior COPD (%) | ≥ 10.0 | 3 | 0.88 (0.69-1.11) | 0.290 | 0.0 | 0.501 | 0.673 |
|  | < 10.0 | 1 | 3.17 (0.13-75.28) | 0.475 | - | - |  |
|  | Not reported | 1 | 0.96 (0.69-1.34) | 0.829 | - | - |  |
| Percentage of NYHA III-IV | ≥ 50.0 | 2 | 0.95 (0.77-1.17) | 0.629 | 0.0 | 0.912 | 0.271 |
|  | < 50.0 | 3 | 0.69 (0.40-1.18) | 0.173 | 0.0 | 0.621 |  |
| Valve type | Balloon-expanding | 2 | 0.95 (0.73-1.23) | 0.700 | 0.0 | 0.454 | 0.659 |
|  | Self-expanding | 3 | 0.87 (0.66-1.15) | 0.334 | 0.0 | 0.492 |  |
| Follow-up duration | 30 days | 5 | 0.94 (0.66-1.35) | 0.747 | 0.0 | 0.462 | 0.774 |
|  | 1 year | 4 | 0.84 (0.67-1.06) | 0.143 | 0.0 | 0.661 |  |
|  | 2 years | 3 | 0.93 (0.77-1.14) | 0.502 | 0.0 | 0.775 |  |
| Study quality | High | 3 | 0.92 (0.76-1.12) | 0.417 | 0.0 | 0.556 | 0.675 |
|  | Low | 2 | 0.77 (0.34-1.72) | 0.524 | 0.0 | 0.364 |  |

Table S3. Subgroup analysis for stroke

| Variable | Group | Number of trials | RR and 95% CI | P value | Heterogeneity (%) | P value for heterogeneity | P value between subgroups |
| --- | --- | --- | --- | --- | --- | --- | --- |
| Sample size | ≥ 1000 | 3 | 0.92 (0.73-1.17) | 0.510 | 26.6 | 0.256 | 0.161 |
|  | < 1000 | 4 | 0.61 (0.35-1.08) | 0.092 | 6.3 | 0.362 |  |
| Mean age | ≥ 80.0 | 3 | 1.03 (0.80-1.34) | 0.801 | 0.0 | 0.493 | 0.098 |
|  | < 80.0 | 4 | 0.74 (0.53-1.04) | 0.079 | 19.9 | 0.290 |  |
| STS score | ≥ 3.5 | 3 | 0.88 (0.66-1.18) | 0.404 | 36.9 | 0.205 | 0.583 |
|  | < 3.5 | 4 | 0.71 (0.38-1.35) | 0.302 | 32.2 | 0.219 |  |
| Percentage of DM (%) | ≥ 30.0 | 4 | 0.84 (0.60-1.18) | 0.324 | 55.7 | 0.080 | 0.831 |
|  | < 30.0 | 2 | 0.82 (0.30-2.27) | 0.709 | 0.0 | 0.384 |  |
|  | Not reported | 1 | 0.72 (0.34-1.49) | 0.373 | - | - |  |
| Prior stroke (%) | ≥ 10.0 | 3 | 1.02 (0.80-1.30) | 0.858 | 0.0 | 0.659 | 0.169 |
|  | < 10.0 | 3 | 0.62 (0.27-1.44) | 0.265 | 43.0 | 0.173 |  |
|  | Not reported | 1 | 0.72 (0.34-1.49) | 0.373 | - | - |  |
| Prior PVD (%) | ≥ 10.0 | 2 | 0.91 (0.63-1.31) | 0.608 | 63.3 | 0.099 | 0.688 |
|  | < 10.0 | 4 | 0.71 (0.38-1.35) | 0.302 | 32.2 | 0.219 |  |
|  | Not reported | 1 | 0.72 (0.34-1.49) | 0.373 | - | - |  |
| Prior MI (%) | ≥ 10.0 | 2 | 0.91 (0.63-1.31) | 0.608 | 63.3 | 0.099 | 0.710 |
|  | < 10.0 | 3 | 0.64 (0.31-1.32) | 0.229 | 46.6 | 0.154 |  |
|  | Not reported | 2 | 0.79 (0.39-1.59) | 0.506 | 0.0 | 0.389 |  |
| Prior COPD (%) | ≥ 10.0 | 3 | 1.02 (0.80-1.30) | 0.858 | 0.0 | 0.659 | 0.100 |
|  | < 10.0 | 2 | 0.57 (0.07-4.42) | 0.593 | 59.3 | 0.117 |  |
|  | Not reported | 2 | 0.74 (0.54-1.01) | 0.056 | 0.0 | 0.932 |  |
| Percentage of NYHA III-IV | ≥ 50.0 | 2 | 0.91 (0.63-1.31) | 0.608 | 63.3 | 0.099 | 0.710 |
|  | < 50.0 | 3 | 0.64 (0.31-1.32) | 0.229 | 46.6 | 0.154 |  |
|  | Not reported | 2 | 0.79 (0.39-1.59) | 0.506 | 0.0 | 0.389 |  |
| Valve type | Balloon-expanding | 3 | 0.76 (0.25-2.25) | 0.615 | 62.2 | 0.071 | 0.173 |
|  | Self-expanding | 4 | 0.78 (0.61-1.01) | 0.062 | 0.0 | 0.861 |  |
| Follow-up duration | 30 days | 6 | 0.75 (0.54-1.05) | 0.096 | 29.2 | 0.216 | 0.610 |
|  | 1 year | 4 | 0.90 (0.73-1.11) | 0.348 | 0.0 | 0.730 |  |
|  | 2 years | 4 | 0.89 (0.70-1.12) | 0.317 | 13.3 | 0.326 |  |
| Study quality | High | 4 | 0.84 (0.60-1.18) | 0.324 | 55.7 | 0.080 | 0.571 |
|  | Low | 3 | 0.75 (0.42-1.36) | 0.347 | 0.0 | 0.668 |  |

Table S4. Subgroup analysis for TIA

| Variable | Group | Number of trials | RR and 95% CI | P value | Heterogeneity (%) | P value for heterogeneity | P value between subgroups |
| --- | --- | --- | --- | --- | --- | --- | --- |
| Sample size | ≥ 1000 | 3 | 1.38 (0.99-1.93) | 0.056 | 0.0 | 0.439 | 0.538 |
|  | < 1000 | 2 | 1.99 (0.66-5.99) | 0.223 | 0.0 | 0.757 |  |
| Mean age | ≥ 80.0 | 2 | 1.75 (1.02-2.99) | 0.042 | 0.0 | 0.708 | 0.357 |
|  | < 80.0 | 3 | 1.28 (0.86-1.90) | 0.225 | 0.0 | 0.568 |  |
| STS score | ≥ 3.5 | 2 | 1.51 (1.05-2.19) | 0.027 | 0.0 | 0.541 | 0.522 |
|  | < 3.5 | 3 | 1.19 (0.63-2.25) | 0.591 | 0.0 | 0.513 |  |
| Percentage of DM (%) | ≥ 30.0 | 3 | 1.38 (0.99-1.93) | 0.056 | 0.0 | 0.439 | 0.538 |
|  | < 30.0 | 2 | 1.99 (0.66-5.99) | 0.223 | 0.0 | 0.757 |  |
| Prior stroke (%) | ≥ 10.0 | 3 | 1.45 (0.96-2.20) | 0.081 | 0.0 | 0.399 | 0.900 |
|  | < 10.0 | 2 | 1.39 (0.85-2.28) | 0.188 | 0.0 | 0.606 |  |
| Prior PVD (%) | ≥ 10.0 | 2 | 1.51 (1.05-2.19) | 0.027 | 0.0 | 0.541 | 0.522 |
|  | < 10.0 | 3 | 1.19 (0.63-2.25) | 0.591 | 0.0 | 0.513 |  |
| Prior MI (%) | ≥ 10.0 | 2 | 1.51 (1.05-2.19) | 0.027 | 0.0 | 0.541 | 0.522 |
|  | < 10.0 | 3 | 1.19 (0.63-2.25) | 0.591 | 0.0 | 0.513 |  |
| Prior COPD (%) | ≥ 10.0 | 3 | 1.45 (0.96-2.20) | 0.081 | 0.0 | 0.399 | 0.848 |
|  | < 10.0 | 1 | 3.17 (0.13-75.28) | 0.475 | - | - |  |
|  | Not reported | 1 | 1.36 (0.83-2.24) | 0.223 | - | - |  |
| Percentage of NYHA III-IV | ≥ 50.0 | 2 | 1.51 (1.05-2.19) | 0.027 | 0.0 | 0.541 | 0.522 |
|  | < 50.0 | 3 | 1.19 (0.63-2.25) | 0.591 | 0.0 | 0.513 |  |
| Valve type | Balloon-expanding | 2 | 1.75 (1.02-2.99) | 0.042 | 0.0 | 0.708 | 0.357 |
|  | Self-expanding | 3 | 1.28 (0.86-1.90) | 0.225 | 0.0 | 0.568 |  |
| Follow-up duration | 30 days | 5 | 1.41 (0.79-2.52) | 0.244 | 0.0 | 0.581 | 0.888 |
|  | 1 year | 4 | 1.36 (0.94-1.97) | 0.107 | 0.0 | 0.730 |  |
|  | 2 years | 3 | 1.54 (1.09-2.19) | 0.016 | 0.0 | 0.786 |  |
| Study quality | High | 3 | 1.38 (0.99-1.93) | 0.056 | 0.0 | 0.439 | 0.538 |
|  | Low | 2 | 1.99 (0.66-5.99) | 0.223 | 0.0 | 0.757 |  |

Table S5. Subgroup analysis for post-procedural bleeding

| Variable | Group | Number of trials | RR and 95% CI | P value | Heterogeneity (%) | P value for heterogeneity | P value between subgroups |
| --- | --- | --- | --- | --- | --- | --- | --- |
| Sample size | ≥ 1000 | 3 | 0.55 (0.23-1.36) | 0.197 | 96.8 | <0.001 | 0.210 |
|  | < 1000 | 3 | 0.58 (0.44-0.76) | <0.001 | 0.0 | 0.868 |  |
| Mean age | ≥ 80.0 | 3 | 0.46 (0.29-0.72) | 0.001 | 73.6 | 0.023 | <0.001 |
|  | < 80.0 | 3 | 0.64 (0.26-1.54) | 0.317 | 92.1 | <0.001 |  |
| STS score | ≥ 3.5 | 3 | 0.65 (0.29-1.44) | 0.290 | 96.8 | <0.001 | 0.407 |
|  | < 3.5 | 3 | 0.42 (0.30-0.60) | <0.001 | 0.0 | 0.441 |  |
| Percentage of DM (%) | ≥ 30.0 | 3 | 0.55 (0.23-1.36) | 0.197 | 96.8 | <0.001 | 0.444 |
|  | < 30.0 | 2 | 0.55 (0.31-0.95) | 0.034 | 0.0 | 0.629 |  |
|  | Not reported | 1 | 0.59 (0.43-0.81) | 0.001 | - | - |  |
| Prior stroke (%) | ≥ 10.0 | 3 | 0.37 (0.32-0.43) | <0.001 | 0.0 | 0.432 | <0.001 |
|  | < 10.0 | 2 | 1.30 (0.99-1.73) | 0.063 | 0.0 | 0.880 |  |
|  | Not reported | 1 | 0.59 (0.43-0.81) | 0.001 | - | - |  |
| Prior PVD (%) | ≥ 10.0 | 2 | 0.68 (0.19-2.41) | 0.555 | 98.4 | <0.001 | 0.405 |
|  | < 10.0 | 3 | 0.42 (0.30-0.60) | <0.001 | 0.0 | 0.441 |  |
|  | Not reported | 1 | 0.59 (0.43-0.81) | 0.001 | - | - |  |
| Prior MI (%) | ≥ 10.0 | 2 | 0.68 (0.19-2.41) | 0.555 | 98.4 | <0.001 | 0.355 |
|  | < 10.0 | 2 | 0.42 (0.28-0.62) | <0.001 | 16.6 | 0.274 |  |
|  | Not reported | 2 | 0.59 (0.43-0.82) | 0.001 | 0.0 | 0.675 |  |
| Prior COPD (%) | ≥ 10.0 | 3 | 0.37 (0.32-0.43) | <0.001 | 0.0 | 0.432 | <0.001 |
|  | < 10.0 | 1 | 1.06 (0.07-16.27) | 0.967 | - | - |  |
|  | Not reported | 2 | 0.88 (0.40-1.93) | 0.751 | 92.6 | <0.001 |  |
| Percentage of NYHA III-IV | ≥ 50.0 | 2 | 0.68 (0.19-2.41) | 0.555 | 98.4 | <0.001 | 0.355 |
|  | < 50.0 | 2 | 0.42 (0.28-0.62) | <0.001 | 16.6 | 0.274 |  |
|  | Not reported | 2 | 0.59 (0.43-0.82) | 0.001 | 0.0 | 0.675 |  |
| Valve type | Balloon-expanding | 2 | 0.36 (0.31-0.42) | <0.001 | 0.0 | 0.442 | <0.001 |
|  | Self-expanding | 4 | 0.63 (0.35-1.14) | 0.128 | 89.5 | <0.001 |  |
| Follow-up duration | 30 days | 5 | 0.51 (0.21-1.28) | 0.154 | 95.9 | <0.001 | 0.118 |
|  | 1 year | 2 | 0.33 (0.29-0.39) | <0.001 | 0.0 | 0.795 |  |
|  | 2 years | 2 | 0.45 (0.28-0.73) | 0.001 | 85.9 | 0.008 |  |
| Study quality | High | 3 | 0.55 (0.23-1.36) | 0.197 | 96.8 | <0.001 | 0.210 |
|  | Low | 3 | 0.58 (0.44-0.76) | <0.001 | 0.0 | 0.868 |  |

Table S6. Subgroup analysis for permanent pacemarker implatation

| Variable | Group | Number of trials | RR and 95% CI | P value | Heterogeneity (%) | P value for heterogeneity | P value between subgroups |
| --- | --- | --- | --- | --- | --- | --- | --- |
| Sample size | ≥ 1000 | 3 | 2.38 (1.14-4.94) | 0.020 | 95.0 | <0.001 | 0.028 |
|  | < 1000 | 3 | 4.41 (1.50-12.95) | 0.007 | 71.5 | 0.030 |  |
| Mean age | ≥ 80.0 | 3 | 1.80 (0.86-3.75) | 0.118 | 77.8 | 0.011 | <0.001 |
|  | < 80.0 | 3 | 4.09 (2.58-6.50) | <0.001 | 74.0 | 0.021 |  |
| STS score | ≥ 3.5 | 3 | 2.34 (1.03-5.31) | 0.043 | 94.8 | <0.001 | 0.010 |
|  | < 3.5 | 3 | 4.43 (1.58-12.43) | 0.005 | 73.0 | 0.025 |  |
| Percentage of DM (%) | ≥ 30.0 | 3 | 2.38 (1.14-4.94) | 0.020 | 95.0 | <0.001 | 0.006 |
|  | < 30.0 | 2 | 6.86 (1.77-26.58) | 0.005 | 34.6 | 0.216 |  |
|  | Not reported | 1 | 2.79 (1.70-4.56) | <0.001 | - | - |  |
| Prior stroke (%) | ≥ 10.0 | 3 | 2.99 (1.19-7.51) | 0.020 | 94.2 | <0.001 | <0.001 |
|  | < 10.0 | 2 | 3.86 (2.91-5.11) | <0.001 | 0.0 | 0.615 |  |
|  | Not reported | 1 | 2.79 (1.70-4.56) | <0.001 | - | - |  |
| Prior PVD (%) | ≥ 10.0 | 2 | 2.16 (0.68-6.90) | 0.195 | 97.3 | <0.001 | 0.019 |
|  | < 10.0 | 3 | 4.43 (1.58-12.43) | 0.005 | 73.0 | 0.025 |  |
|  | Not reported | 1 | 2.79 (1.70-4.56) | <0.001 | - | - |  |
| Prior MI (%) | ≥ 10.0 | 2 | 2.16 (0.68-6.90) | 0.195 | 97.3 | <0.001 | 0.019 |
|  | < 10.0 | 2 | 5.09 (1.45-17.86) | 0.011 | 86.2 | 0.007 |  |
|  | Not reported | 2 | 2.76 (1.70-4.46) | <0.001 | 0.0 | 0.823 |  |
| Prior COPD (%) | ≥ 10.0 | 3 | 2.99 (1.19-7.51) | 0.020 | 94.2 | <0.001 | <0.001 |
|  | < 10.0 | 1 | 2.12 (0.20-22.30) | 0.532 | - | - |  |
|  | Not reported | 2 | 3.51 (2.59-4.75) | <0.001 | 25.1 | 0.248 |  |
| Percentage of NYHA III-IV | ≥ 50.0 | 2 | 2.16 (0.68-6.90) | 0.195 | 97.3 | <0.001 | 0.019 |
|  | < 50.0 | 2 | 5.09 (1.45-17.86) | 0.011 | 86.2 | 0.007 |  |
|  | Not reported | 2 | 2.76 (1.70-4.46) | <0.001 | 0.0 | 0.823 |  |
| Valve type | Balloon-expanding | 2 | 1.21 (0.93-1.56) | 0.149 | 0.0 | 0.638 | <0.001 |
|  | Self-expanding | 4 | 3.67 (2.57-5.25) | <0.001 | 65.2 | 0.035 |  |
| Follow-up duration | 30 days | 5 | 3.16 (1.62-6.16) | 0.001 | 89.8 | <0.001 | 0.004 |
|  | 1 year | 3 | 3.17 (1.19-8.45) | 0.021 | 94.1 | <0.001 |  |
|  | 2 years | 3 | 3.01 (1.04-8.72) | 0.042 | 92.8 | <0.001 |  |
| Study quality | High | 3 | 2.38 (1.14-4.94) | 0.020 | 95.0 | <0.001 | 0.028 |
|  | Low | 3 | 4.41 (1.50-12.95) | 0.007 | 71.5 | 0.030 |  |

Table S7. Subgroup analysis for new-onset or worsening atrial fibrillation

| Variable | Group | Number of trials | RR and 95% CI | P value | Heterogeneity (%) | P value for heterogeneity | P value between subgroups |
| --- | --- | --- | --- | --- | --- | --- | --- |
| Sample size | ≥ 1000 | 3 | 0.32 (0.24-0.41) | <0.001 | 78.6 | 0.009 | 1.000 |
|  | < 1000 | 3 | 0.32 (0.13-0.80) | 0.015 | 94.8 | <0.001 |  |
| Mean age | ≥ 80.0 | 2 | 0.51 (0.32-0.84) | 0.007 | 85.0 | 0.010 | <0.001 |
|  | < 80.0 | 4 | 0.25 (0.18-0.35) | <0.001 | 81.5 | 0.001 |  |
| STS score | ≥ 3.5 | 3 | 0.42 (0.28-0.64) | <0.001 | 89.5 | <0.001 | <0.001 |
|  | < 3.5 | 3 | 0.23 (0.14-0.40) | <0.001 | 86.7 | 0.001 |  |
| Percentage of DM (%) | ≥ 30.0 | 4 | 0.26 (0.18-0.37) | <0.001 | 88.1 | <0.001 | <0.001 |
|  | < 30.0 | 1 | 0.37 (0.27-0.52) | <0.001 | - | - |  |
|  | Not reported | 1 | 0.67 (0.49-0.92) | 0.014 | - | - |  |
| Prior stroke (%) | ≥ 10.0 | 3 | 0.34 (0.25-0.46) | <0.001 | 77.2 | 0.013 | <0.001 |
|  | < 10.0 | 2 | 0.20 (0.09-0.46) | <0.001 | 91.8 | <0.001 |  |
|  | Not reported | 1 | 0.67 (0.49-0.92) | 0.014 | - | - |  |
| Prior PVD (%) | ≥ 10.0 | 2 | 0.35 (0.25-0.47) | <0.001 | 79.8 | 0.026 | <0.001 |
|  | < 10.0 | 3 | 0.23 (0.14-0.40) | <0.001 | 86.7 | 0.001 |  |
|  | Not reported | 1 | 0.67 (0.49-0.92) | 0.014 | - | - |  |
| Prior MI (%) | ≥ 10.0 | 2 | 0.35 (0.25-0.47) | <0.001 | 79.8 | 0.026 | <0.001 |
|  | < 10.0 | 3 | 0.23 (0.14-0.40) | <0.001 | 86.7 | 0.001 |  |
|  | Not reported | 1 | 0.67 (0.49-0.92) | 0.014 | - | - |  |
| Prior COPD (%) | ≥ 10.0 | 3 | 0.34 (0.25-0.46) | <0.001 | 77.2 | 0.013 | <0.001 |
|  | < 10.0 | 1 | 0.13 (0.08-0.20) | <0.001 | - | - |  |
|  | Not reported | 2 | 0.44 (0.20-0.98) | 0.045 | 94.6 | <0.001 |  |
| Percentage of NYHA III-IV | ≥ 50.0 | 2 | 0.35 (0.25-0.47) | <0.001 | 79.8 | 0.026 | <0.001 |
|  | < 50.0 | 3 | 0.23 (0.14-0.40) | <0.001 | 86.7 | 0.001 |  |
|  | Not reported | 1 | 0.67 (0.49-0.92) | 0.014 | - | - |  |
| Valve type | Balloon-expanding | 2 | 0.23 (0.07-0.73) | 0.013 | 95.6 | <0.001 | 1.000 |
|  | Self-expanding | 4 | 0.37 (0.25-0.53) | <0.001 | 88.0 | <0.001 |  |
| Follow-up duration | 30 days | 5 | 0.25 (0.19-0.33) | <0.001 | 79.7 | 0.001 | <0.001 |
|  | 1 year | 3 | 0.32 (0.25-0.41) | <0.001 | 64.1 | 0.062 |  |
|  | 2 years | 3 | 0.46 (0.33-0.64) | <0.001 | 75.2 | 0.018 |  |
| Study quality | High | 4 | 0.26 (0.18-0.37) | <0.001 | 88.1 | <0.001 | <0.001 |
|  | Low | 2 | 0.50 (0.28-0.89) | 0.018 | 83.6 | 0.014 |  |

Table S8. Subgroup analysis for acute kidney injury

| Variable | Group | Number of trials | RR and 95% CI | P value | Heterogeneity (%) | P value for heterogeneity | P value between subgroups |
| --- | --- | --- | --- | --- | --- | --- | --- |
| Sample size | ≥ 1000 | 3 | 0.44 (0.26-0.73) | 0.002 | 52.1 | 0.124 | 0.224 |
|  | < 1000 | 3 | 0.32 (0.09-1.12) | 0.075 | 37.2 | 0.203 |  |
| Mean age | ≥ 80.0 | 3 | 0.52 (0.27-1.01) | 0.052 | 50.3 | 0.134 | 0.055 |
|  | < 80.0 | 3 | 0.30 (0.18-0.50) | <0.001 | 0.0 | 0.568 |  |
| STS score | ≥ 3.5 | 3 | 0.43 (0.26-0.71) | 0.001 | 58.2 | 0.092 | 0.354 |
|  | < 3.5 | 3 | 0.33 (0.09-1.22) | 0.097 | 37.5 | 0.202 |  |
| Percentage of DM (%) | ≥ 30.0 | 3 | 0.44 (0.26-0.73) | 0.002 | 52.1 | 0.124 | 0.474 |
|  | < 30.0 | 2 | 0.46 (0.02-12.90) | 0.648 | 68.5 | 0.075 |  |
|  | Not reported | 1 | 0.32 (0.16-0.64) | 0.001 | - | - |  |
| Prior stroke (%) | ≥ 10.0 | 3 | 0.41 (0.19-0.89) | 0.023 | 55.1 | 0.108 | 0.297 |
|  | < 10.0 | 2 | 0.58 (0.08-4.30) | 0.596 | 49.0 | 0.162 |  |
|  | Not reported | 1 | 0.32 (0.16-0.64) | 0.001 | - | - |  |
| Prior PVD (%) | ≥ 10.0 | 2 | 0.47 (0.24-0.93) | 0.031 | 69.2 | 0.072 | 0.302 |
|  | < 10.0 | 3 | 0.33 (0.09-1.22) | 0.097 | 37.5 | 0.202 |  |
|  | Not reported | 1 | 0.32 (0.16-0.64) | 0.001 | - | - |  |
| Prior MI (%) | ≥ 10.0 | 2 | 0.47 (0.24-0.93) | 0.031 | 69.2 | 0.072 | 0.276 |
|  | < 10.0 | 2 | 0.27 (0.11-0.66) | 0.004 | 8.6 | 0.296 |  |
|  | Not reported | 2 | 0.59 (0.08-4.28) | 0.599 | 48.2 | 0.165 |  |
| Prior COPD (%) | ≥ 10.0 | 3 | 0.41 (0.19-0.89) | 0.023 | 55.1 | 0.108 | 0.112 |
|  | < 10.0 | 1 | 3.17 (0.13-75.28) | 0.475 | - | - |  |
|  | Not reported | 2 | 0.32 (0.20-0.51) | <0.001 | 0.0 | 0.979 |  |
| Percentage of NYHA III-IV | ≥ 50.0 | 2 | 0.47 (0.24-0.93) | 0.031 | 69.2 | 0.072 | 0.276 |
|  | < 50.0 | 2 | 0.27 (0.11-0.66) | 0.004 | 8.6 | 0.296 |  |
|  | Not reported | 2 | 0.59 (0.08-4.28) | 0.599 | 48.2 | 0.165 |  |
| Valve type | Balloon-expanding | 2 | 0.65 (0.44-0.98) | 0.040 | 0.0 | 0.325 | 0.010 |
|  | Self-expanding | 4 | 0.31 (0.20-0.46) | <0.001 | 0.0 | 0.765 |  |
| Follow-up duration | 30 days | 5 | 0.35 (0.24-0.52) | <0.001 | 0.0 | 0.453 | 0.164 |
|  | 1 year | 2 | 0.52 (0.27-1.01) | 0.055 | 52.1 | 0.149 |  |
|  | 2 years | 2 | 0.48 (0.25-0.93) | 0.031 | 64.6 | 0.093 |  |
| Study quality | High | 3 | 0.44 (0.26-0.73) | 0.002 | 52.1 | 0.124 | 0.224 |
|  | Low | 3 | 0.32 (0.09-1.12) | 0.075 | 37.2 | 0.203 |  |

Table S9. Subgroup analysis for major vascular complications

| Variable | Group | Number of trials | RR and 95% CI | P value | Heterogeneity (%) | P value for heterogeneity | P value between subgroups |
| --- | --- | --- | --- | --- | --- | --- | --- |
| Sample size | ≥ 1000 | 3 | 1.19 (0.87-1.63) | 0.281 | 63.3 | 0.066 | 0.010 |
|  | < 1000 | 2 | 3.63 (1.50-8.76) | 0.004 | 0.0 | 0.968 |  |
| Mean age | ≥ 80.0 | 2 | 2.01 (0.96-4.21) | 0.062 | 51.2 | 0.152 | 0.007 |
|  | < 80.0 | 3 | 1.08 (0.78-1.50) | 0.637 | 29.1 | 0.244 |  |
| STS score | ≥ 3.5 | 3 | 1.46 (0.88-2.40) | 0.142 | 79.5 | 0.008 | 0.843 |
|  | < 3.5 | 2 | 1.61 (0.51-5.08) | 0.412 | 56.1 | 0.131 |  |
| Percentage of DM (%) | ≥ 30.0 | 3 | 1.19 (0.87-1.63) | 0.281 | 63.3 | 0.066 | 0.036 |
|  | < 30.0 | 1 | 3.72 (0.81-17.23) | 0.092 | - | - |  |
|  | Not reported | 1 | 3.58 (1.22-10.52) | 0.020 | - | - |  |
| Prior stroke (%) | ≥ 10.0 | 3 | 1.45 (0.98-2.16) | 0.064 | 32.4 | 0.228 | 0.010 |
|  | < 10.0 | 1 | 1.00 (0.82-1.23) | 0.983 | - | - |  |
|  | Not reported | 1 | 3.58 (1.22-10.52) | 0.020 | - | - |  |
| Prior PVD (%) | ≥ 10.0 | 2 | 1.23 (0.79-1.93) | 0.354 | 81.5 | 0.020 | 0.112 |
|  | < 10.0 | 2 | 1.61 (0.51-5.08) | 0.412 | 56.1 | 0.131 |  |
|  | Not reported | 1 | 3.58 (1.22-10.52) | 0.020 | - | - |  |
| Prior MI (%) | ≥ 10.0 | 2 | 1.23 (0.79-1.93) | 0.354 | 81.5 | 0.020 | 0.112 |
|  | < 10.0 | 2 | 1.61 (0.51-5.08) | 0.412 | 56.1 | 0.131 |  |
|  | Not reported | 1 | 3.58 (1.22-10.52) | 0.020 | - | - |  |
| Prior COPD (%) | ≥ 10.0 | 3 | 1.45 (0.98-2.16) | 0.064 | 32.4 | 0.228 | 0.049 |
|  | Not reported | 2 | 1.69 (0.49-5.82) | 0.404 | 80.9 | 0.022 |  |
| Percentage of NYHA III-IV | ≥ 50.0 | 2 | 1.23 (0.79-1.93) | 0.354 | 81.5 | 0.020 | 0.112 |
|  | < 50.0 | 2 | 1.61 (0.51-5.08) | 0.412 | 56.1 | 0.131 |  |
|  | Not reported | 1 | 3.58 (1.22-10.52) | 0.020 | - | - |  |
| Valve type | Balloon-expanding | 1 | 1.58 (1.14-2.19) | 0.006 | - | - | 0.041 |
|  | Self-expanding | 4 | 1.41 (0.84-2.36) | 0.195 | 62.0 | 0.048 |  |
| Follow-up duration | 30 days | 4 | 2.22 (1.15-4.30) | 0.017 | 76.7 | 0.005 | 0.013 |
|  | 1 year | 3 | 1.21 (0.90-1.61) | 0.206 | 51.8 | 0.126 |  |
|  | 2 years | 3 | 1.46 (0.88-2.40) | 0.142 | 79.5 | 0.008 |  |
| Study quality | High | 3 | 1.19 (0.87-1.63) | 0.281 | 63.3 | 0.066 | 0.010 |
|  | Low | 2 | 3.63 (1.50-8.76) | 0.004 | 0.0 | 0.968 |  |

Table S10. The results of previous meta-analyses

| Study | Included studies | Population | Mortality | Cardiac death | Stroke | TIA | PPB | PPI | NOWAF | AKI | Major vascular complications | MI | Valvular endocarditis | Aortic-valve reintervention | Coronary obstruction | Cardiogenic shock |
| --- | --- | --- | --- | --- | --- | --- | --- | --- | --- | --- | --- | --- | --- | --- | --- | --- |
| Arora [31] | 1 RCT and 3 PSM cohort studies | Low surgical risk | Reduced | Not reported | Reduced | Not reported | Reduced | Increased | Not reported | Reduced | Increased | Not reported | Not reported | Increased | Not reported | Not reported |
| Singh [32] | 3 RCTs and 5 PSM cohort studies | Intermediate surgical risk | Reduced | Not significant | Not significant | Not reported | Not reported | Increased | Not reported | Not reported | Not reported | Not reported | Not reported | Increased | Not reported | Not reported |
| Khan [33] | 1 RCT and 5 observational studies | Intermediate surgical risk | Not significant | Not reported | Increased | Not reported | Increased | Increased | Not reported | Not reported | Increased | Not reported | Not reported | Not reported | Not reported | Not reported |
| Khan [34] | 4 RCTs and 8 PSM studies | Low to intermediate surgical risk | Not significant | Not reported | Not reported | Not reported | Not reported | Increased | Reduced | Reduced | Not reported | Not reported | Not reported | Increased | Not reported | Not reported |
| Tam [35] | 4 RCTs and 9 PSM studies | Low to intermediate surgical risk | Not significant | Not reported | Reduced | Not reported | Reduced | Increased | Reduced | Reduced | Not reported | Not reported | Not reported | Not reported | Not reported | Reduced |
| Elmaraezy [36] | 4 RCTs, 5 prospective, and 2 retrospective studies | Low to intermediate surgical risk | Not significant | Not reported | Not significant | Not reported | Reduced | Increased | Not reported | Reduced | Increased | Not significant | Not reported | Not reported | Not reported | Not reported |
| Garg [37] | 5 RCTs and 5 observational studies | Low to intermediate surgical risk | Not significant | Not reported | Not significant | Not reported | Reduced | Increased | Reduced | Reduced | Increased | Not significant | Not reported | Not reported | Not reported | Not reported |
| Zhou [38] | 3 RCTs and 4 cohort studies | Low to intermediate surgical risk | Not significant | Not significant | Reduced | Not reported | Not reported | Increased | Not reported | Not reported | Increased | Reduced | Not reported | Not reported | Not reported | Not reported |
| Ando [39] | 7 RCTs | Low to intermediate surgical risk | Not significant | Not reported | Reduced | Not reported | Not reported | Not reported | Not reported | Not reported | Not reported | Not reported | Not reported | Not reported | Not reported | Not reported |
